# Diagnostic Value of Comprehensive Echocardiographic Assessment Including Speckle-Tracking in Patients with Sarcoidosis Versus Healthy Controls: A Systematic Review and Meta-Analysis

**DOI:** 10.3390/diagnostics15060708

**Published:** 2025-03-12

**Authors:** Hritvik Jain, Maryam Shahzad, Muneeba Ahsan, Rahul Patel, Jagjot Singh, Ramez M. Odat, Aman Goyal, Raveena Kelkar, Nishad Barve, Hina Farrukh, Raheel Ahmed

**Affiliations:** 1 Department of Cardiology, All India Institute of Medical Sciences Jodhpur, Jodhpur 342005, India; hritvikjain2001@gmail.com; 2 Department of Internal Medicine, Dow University of Health Sciences, Karachi 74200, Pakistan; maryamshahzad575@gmail.com (M.S.); muneeba2003ahsan@gmail.com (M.A.); 3 Department of Internal Medicine, University of North Carolina Blue Ridge Hospital, Morganton, NC 28655, USA; rahul.patel@unchealth.unc.edu; 4 Department of Internal Medicine, Government Medical College Amritsar, Amritsar 143001, India; ijagjot@gmail.com; 5 Department of Internal Medicine, Faculty of Medicine, Jordan University of Science and Technology, Irbid 22110, Jordan; rmodat22@med.just.edu.jo; 6 Department of Internal Medicine, King Edward Memorial Hospital and Seth G.S. Medical College, Mumbai 400012, India; amanmgy@gmail.com; 7 Department of Critical Care Medicine, Alchemist Hospitals Panchkula, Panchkula 134112, India; 8 Department of Internal Medicine, Wentworth-Douglass Hospital, Dover, NH 03820, USA; raveenakelkar02@gmail.com; 9 Department of Internal Medicine, Lowell General Hospital, Lowell, MA 01854, USA; nishad.barve2007@gmail.com; 10 Department of Internal Medicine, University of Florida Health—Central Florida, The Villages, FL 32608, USA; hinafarrukh.hf@gmail.com; 11 Department of Cardiology, National Heart and Lung Institute, Imperial College London, London SW7 2AZ, UK

**Keywords:** sarcoidosis, echocardiography, doppler echocardiography, speckle tracking echocardiography, cardiac sarcoidosis, meta-analysis

## Abstract

**Background**: Cardiac involvement in sarcoidosis is often subclinical, with late manifestations associated with poorer prognosis. Speckle-tracking echocardiography (STE) is gaining attention due to its ability to detect subclinical alterations in myocardial contraction patterns and quantification of abnormal parameters. **Methods**: Databases, including PubMed, Cochrane Central, Embase, Scopus, and Web of Science, were searched to identify studies comparing echocardiographic parameters in sarcoidosis patients with healthy controls. Mean difference (MD) with 95% confidence intervals (CI) were pooled using the inverse-variance random-effects model in Review Manager Version 5.4.1. Statistical significance was considered at *p*-value <0.05. **Results**: Thirteen studies with 1416 participants (854—sarcoidosis; 562—healthy controls) were included. In a pooled analysis, patients with sarcoidosis demonstrated a significantly lower left ventricular global longitudinal strain (LV GLS) (Mean Difference [MD]: −3.60; 95% Confidence Interval [CI]: −4.76, −2.43; *p* < 0.0001) and left ventricular global circumferential strain (LV GCS) (MD: −2.52; 95% CI: −4.61, −0.43; *p* = 0.02), along with a significantly higher pulmonary artery systolic pressure (PASP) (MD: 4.19; 95% CI: 0.08, 8.29; *p* = 0.05), left ventricular end-systolic diameter (LVESD) (MD: 0.90; 95% CI: 0.10, 1.71; *p* = 0.03), A-wave velocity (MD: 3.36; 95% CI: 0.33, 6.39; *p* = 0.03), and E/E’ ratio (MD: 1.33; 95% CI: 0.42, 2.23; *p* = 0.004) compared to healthy controls. No significant differences were noted in left ventricular ejection fraction (LVEF), left ventricular global radial strain (LV GRS), interventricular septal thickness (IVST), tricuspid annular plane systolic excursion (TAPSE), left ventricular end-diastolic diameter (LVEDD), E-wave velocity, and E/A ratio. **Conclusions**: STE serves as a promising imaging modality in detecting subclinical cardiac involvement in sarcoidosis patients with no overt cardiac manifestations. A widespread cardiovascular evaluation of sarcoidosis patients with STE is recommended to detect these altered myocardial contractile patterns. The early detection of cardiac sarcoidosis is essential to prevent adverse clinical outcomes and improve mortality.

## 1. Introduction

Sarcoidosis is a systemic inflammatory disease characterized by the formation of non-caseating granulomas in various organ systems, leading to significant morbidity and mortality [1]. In patients with systemic sarcoidosis, the prevalence of cardiac involvement may range from 3% to as high as 60%. Approximately one-third of patients with systemic sarcoidosis may have subclinical cardiac involvement, and the condition is often underdiagnosed due to the absence of clinical symptoms [2,3]. Even though the prevalence of cardiac sarcoidosis (CS) is low, it may lead to a variety of life-threatening conditions, including atrioventricular block, arrhythmias, heart failure (HF), and sudden cardiac death (SCD). The clinical presentation can range from asymptomatic findings to SCD [4]. The overall five-year survival in sarcoidosis is 60%, and approximately 85% of deaths are attributable to CS [5,6]. Considering that cardiac involvement is one of the leading causes of mortality in sarcoidosis, it is clinically important to diagnose and manage early to prevent adverse cardiovascular complications.

The initial screening for CS is challenging due to its often-asymptomatic nature in many individuals. Patients with CS may not exhibit symptoms until advanced disease stages, leading to a delay in diagnosis. Moreover, while advanced imaging techniques, like cardiac magnetic resonance (CMR) imaging and positron emission tomography (PET), are highly sensitive for detecting cardiac involvement in sarcoidosis, these modalities are expensive and not universally accessible [7,8,9]. Endomyocardial biopsy (EMB) is the gold standard for diagnosis, but it has limited sensitivity due to the patchy distribution of granulomas within the myocardium [10,11].

Given these challenges, transthoracic echocardiography (TTE) has emerged as a potential first-line screening tool due to its non-invasive nature, accessibility, and cost-effectiveness. However, the utility of TTE in detecting CS remains controversial, as the echocardiographic findings in sarcoidosis patients are not yet well-defined. Structural changes, such as left ventricular (LV) dysfunction, wall motion abnormalities, and right ventricular (RV) involvement, have been reported in various studies, but no consistent echocardiographic criteria for diagnosing CS on echocardiography exist [12,13]. Speckle-tracking echocardiography (STE) is a recently developed imaging technique that can quantify and measure myocardial deformation [14]. Through STE, subclinical myocardial deformation abnormalities can be detected before overt clinical manifestations develop [15,16,17].

This systematic review and meta-analysis aims to assess the role of STE in detecting subclinical cardiac involvement in patients with sarcoidosis compared to healthy controls, while also evaluating conventional echocardiographic parameters.

## 2. Methods

This systematic review and meta-analysis followed the PRISMA 2020 guidelines and the suggested procedures of the Cochrane Collaboration (Supplementary Table S1). The protocol for the systematic review was prospectively registered in the PROSPERO International Prospective Register of Systematic Reviews under the unique identifier code CRD42024621279.

### 2.1. Data Sources and Search Strategy

A comprehensive systematic review of the major bibliographic databases, including MEDLINE (via PubMed), COCHRANE Central, Embase, Scopus, and Web of Science, was carried out through December 2024. The search aimed to retrieve studies evaluating echocardiographic findings of patients with sarcoidosis compared to healthy controls. No restrictions on language or publication year were imposed. To create a search string, keywords, such as ‘sarcoidosis’, ‘echocardiography’, ‘myocardial deformation’, ‘Doppler echocardiography’, and ‘speckle tracking’, were combined with ‘AND ‘OR’ Boolean operators. To ensure completeness, the reference lists of the included studies were manually reviewed to identify additional studies. The complete database-specific search strategies are detailed in Supplementary Table S2.

### 2.2. Eligibility Criteria

This systematic review evaluated studies using the standard PICOS framework (population, intervention, comparison, outcomes, and study designs). Studies were included if they (a) were randomized controlled trials, case-control, or cohort studies, (b) included patients with confirmed sarcoidosis in one arm, (c) included healthy controls in one arm, (d) performed echocardiography for all participants, and (e) reported one of the parameters of interest.

The studies were excluded if they (a) were not randomized controlled trials, or case-control, or cohort studies, (b) did not assess the role of STE in detecting subclinical cardiac involvement by comparing sarcoidosis patients without known CS to healthy controls, or (c) did not report our relevant echocardiographic parameters.

### 2.3. Data

The data of interest included LV global longitudinal strain (LV GLS), LV global circumferential strain (LV GCS), LV ejection fraction (LVEF), LV global radial strain (LV GRS), interventricular septal thickness (IVST), tricuspid annular plane systolic excursion (TAPSE), pulmonary artery systolic pressure (PASP), LV end-diastolic diameter (LVEDD), LV end-systolic diameter (LVESD), E-wave velocity, A-wave velocity, E/A ratio, and E/E’ ratio.

### 2.4. Study Selection

All retrieved records from the systematic database search were imported into the EndNote Reference Manager (Version X7.5) (Clarivate Analytics, Philadelphia, PA, USA). The entire selection of records underwent the removal of duplicates. Two independent reviewers (H.J. and M.S.) conducted preliminary screening using titles and abstracts to include relevant studies. Potentially relevant studies were considered eligible for a comprehensive full-text evaluation for inclusion. Any disagreements were resolved through consensus or by consulting a third independent reviewer (R.A.).

### 2.5. Data Extraction and Quality Assessment

Data extraction was independently performed by two investigators (H.J. and M.A.) using a predesigned Microsoft Excel spreadsheet. Any discrepancies were addressed by either mutual consensus or by a third investigator (M.S.). The extracted data included the author’s name, study design, patient population, number of participants, mean age, females, STE software utilized, organ involvement in sarcoidosis, hypertension, diabetes, smoking, dyslipidemia, and previous history of steroid use. The quality of observational studies was evaluated using the Newcastle–Ottawa Scale (NOS), an eight-item tool designed to assess the quality of non-randomized studies. The NOS assigns scores ranging from 0 to 9, with scores of 7 or higher indicating high quality [18].

### 2.6. Data Analysis

The data synthesis was conducted using Review Manager (RevMan) Version 5.4.1 (Nordic Cochrane Collaboration, Copenhagen, Denmark). Effect estimates were calculated by pooling mean differences (MDs) with 95% confidence intervals (CIs) using the inverse-variance random-effects model. Study heterogeneity was estimated using the Higgins I^2^ metric, where values < 50% represent low, 50–75% moderate, and >75% represent high heterogeneity [19]. To identify studies significantly influencing heterogeneity, a leave-one-out analysis was conducted by sequentially excluding one study to evaluate its influence on overall heterogeneity. Publication bias assessment was carried out by the visual inspection of funnel plots, with asymmetry indicating its presence [20]. All statistical significance was considered at a *p*-value of less than 0.05.

## 3. Results

**Figure 1:**
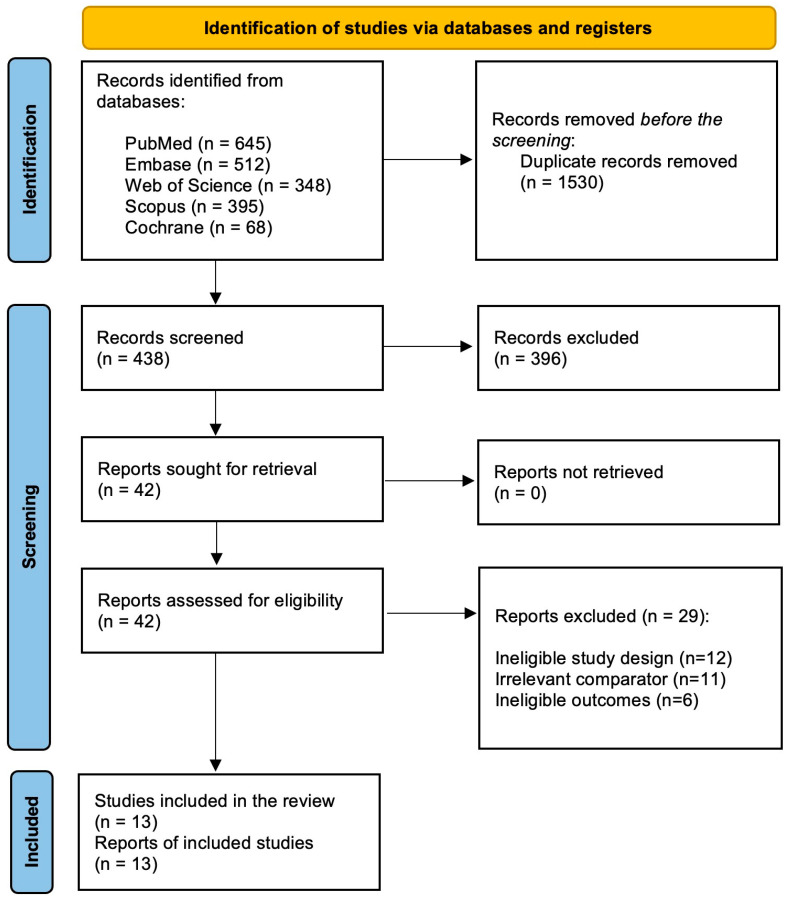
PRISMA flowchart depicting the study selection and screening process.

The initial database search retrieved 1968 records. After removing duplicates (*n* = 1530), 438 records were subjected to preliminary screening using titles and abstracts. During the preliminary screening, 396 records were excluded. The remaining 42 records underwent a comprehensive full-text review against the predefined eligibility criteria. During the full-text review, 29 records were excluded due to various reasons: ineligible study design (*n* = 12), irrelevant comparator (*n* = 11), and ineligible outcomes (*n* = 6). Subsequently, 13 studies were included in the meta-analysis [17,21,22,23,24,25,26,27,28,29,30,31,32]. The study selection process is depicted in Figure 1.

### 3.1. Study and Baseline Characteristics

This meta-analysis included 13 studies, encompassing 1416 participants—854 patients with sarcoidosis and 562 healthy controls [17,21,22,23,24,25,26,27,28,29,30,31,32]. The mean ages of patients and controls were 50.4 ± 11.2 and 49.1 ± 10.8 years, respectively. The included studies were conducted across multiple countries, including Greece [21,25], the United States [17,22], Germany [23], Turkey [24,27,32], the Netherlands [26], Japan [28,29], the Czech Republic [30], and France [31]. The baseline characteristics of the included studies are reported in Table 1. The inclusion and exclusion criteria of each study are reported in Supplementary Table S3.

### 3.2. Echocardiographic Parameters

#### 3.2.1. LV GLS

**Figure 2:**
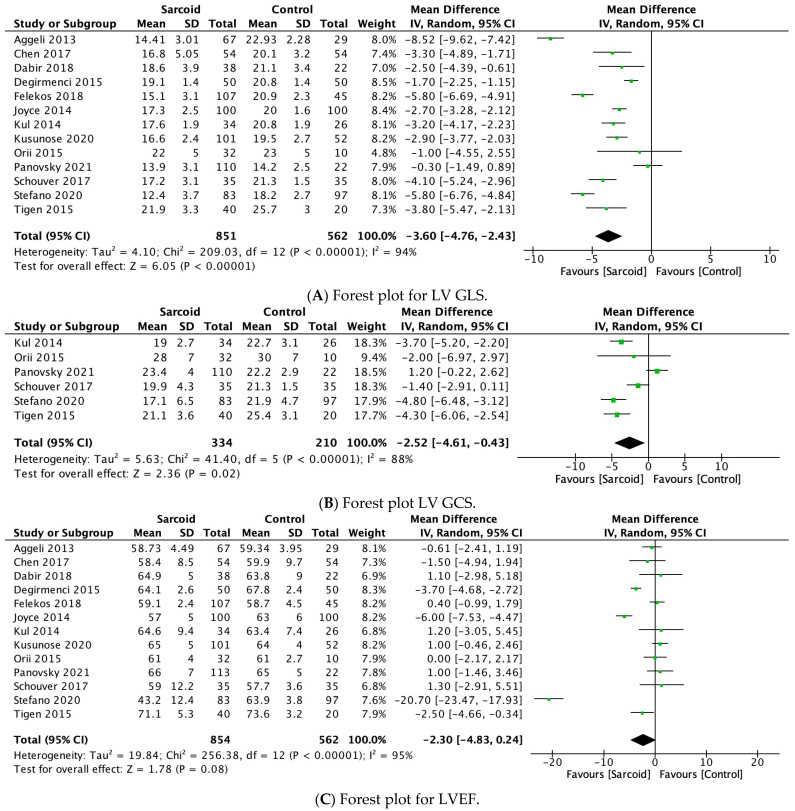

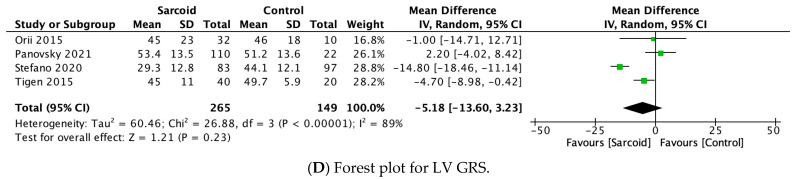
Forest plots comparing echocardiography parameters in patients with sarcoidosis to healthy controls. (**A**) Left ventricular global longitudinal strain (LV GLS); (**B**) left ventricular global circumferential strain (LV GCS); (**C**) left ventricular ejection fraction (LVEF); (**D**) left ventricular global radial strain (LV GRS).

LV GLS was reported by all included studies [17,21,22,23,24,25,26,27,28,29,30,31,32]. The pooled analysis demonstrated a statistically significant reduction in LV GLS in patients with sarcoidosis (MD: −3.60; 95% CI: −4.76, −2.43; *p* < 0.0001; I^2^ = 94%) compared to healthy controls (Figure 2A). High heterogeneity was noted between the studies (I^2^ = 94%), which was reduced to 91% by omitting Aggeli et al., 2023 (Supplementary Figure S1) [21].

#### 3.2.2. LV GCS

LV GCS was reported by six studies [17,27,29,30,31,32]. Patients with sarcoidosis demonstrated a statistically significant reduction in LV GCS (MD: −2.52; 95% CI: −4.61, −0.43; *p* = 0.02; I^2^ = 88%) compared to healthy controls (Figure 2B). High heterogeneity was noted between the studies (I^2^ = 88%), which was reduced to 63% by omitting Panovsky et al., 2021 (Supplementary Figure S2) [30].

#### 3.2.3. LVEF

LVEF was reported by all thirteen studies [17,21,22,23,24,25,26,27,28,29,30,31,32]. No significant difference in LVEF was noted between the two groups (MD: −2.30; 95% CI: −4.83, 0.24; *p* = 0.08; I^2^ = 95%) (Figure 2C). High heterogeneity was noted between the studies (I^2^ = 95%), which was reduced to 87% by omitting Stefano et al., 2020 (Supplementary Figure S3) [17].

#### 3.2.4. LV GRS

LV GRS was reported by four studies [17,29,30,32]. No significant difference in LV GRS was noted between the two groups (MD: −5.18; 95% CI: −13.60, 3.23; *p* = 0.23; I^2^ = 89%) (Figure 2D). High heterogeneity was noted between the studies (I^2^ = 89%), which was reduced to 39% by omitting Stefano et al., 2020 (Supplementary Figure S4) [17].

#### 3.2.5. IVST

**Figure 3:**
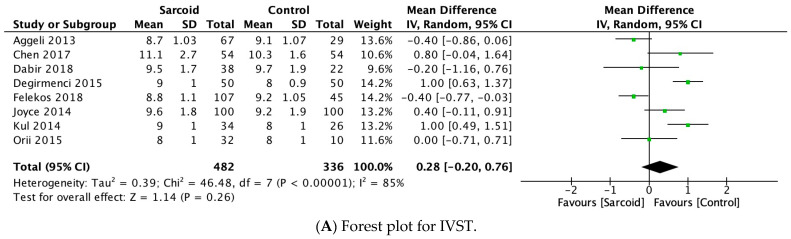

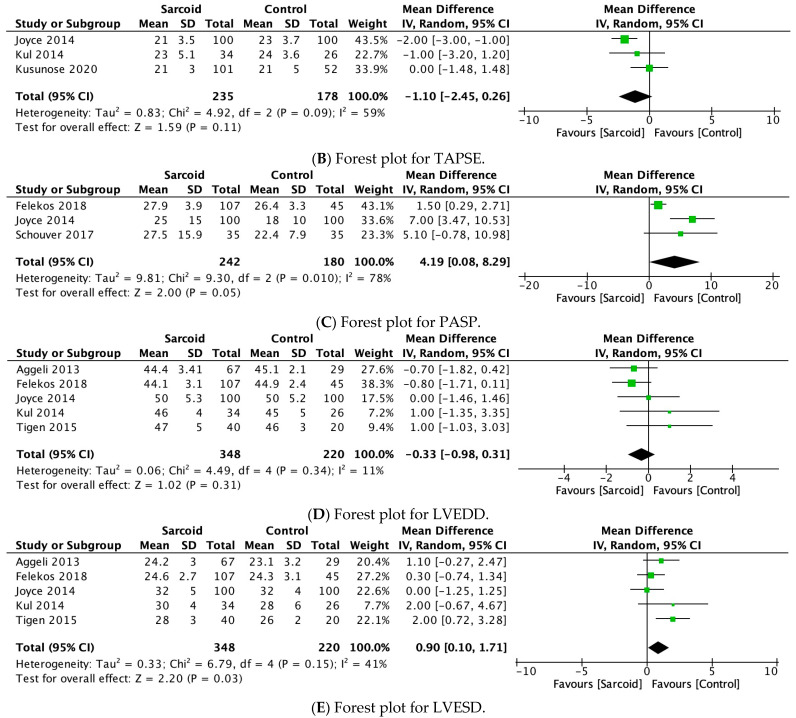
Forest plots comparing echocardiography parameters in patients with sarcoidosis to healthy controls. (**A**) Interventricular septal thickness (IVST); (**B**) tricuspid annular plane systolic excursion (TAPSE); (**C**) pulmonary artery systolic pressure (PASP); (**D**) left ventricular end-diastolic diameter (LVEDD); (**E**) left ventricular end-systolic diameter (LVESD).

IVST was reported by eight studies [21,22,23,24,25,26,27,29]. No significant difference in IVST was noted between the two groups (mm) (MD: 0.28; 95% CI: −0.20, 0.76; *p* = 0.26; I^2^ = 85%) (Figure 3A). The high heterogeneity (I^2^ = 85%) dropped to 78% by omitting Değirmenci et al., 2015 (Supplementary Figure S5) [24].

#### 3.2.6. TAPSE

TAPSE was reported by three studies [26,27,28]. No significant difference in TAPSE was noted between the two groups (MD: −1.10; 95% CI: −2.45, 0.26; *p* = 0.11; I^2^ = 59%) (Figure 3B). The moderate heterogeneity (I^2^ = 59%) dropped to 0% by omitting either Joyce et al., 2015 or Kusunose et al., 2020 (Supplementary Figure S6) [26,28]. However, on omitting Kusunose et al., 2020, the parameter of TAPSE shifted towards a significant reduction in patients with sarcoidosis compared to healthy controls (MD: −1.83; 95% CI: −2.74, −0.92; *p* < 0.0001; I^2^ = 0%) (Supplementary Figure S7).

#### 3.2.7. PASP

PASP was reported by three studies [25,26,31]. On pooled analysis, patients with sarcoidosis demonstrated a significantly higher PASP (MD: 4.19; 95% CI: 0.08, 8.29; *p* = 0.05; I^2^ = 78%) compared to healthy controls (Figure 3C). The high heterogeneity (I^2^ = 78%) dropped to 0% by omitting Felekos et al., 2018 (Supplementary Figure S8) [25].

#### 3.2.8. LVEDD

LVEDD was reported by five studies [21,25,26,27,32]. No significant difference in LVEDD was noted between the two groups (MD: −0.33; 95% CI: −0.98, 0.31; *p* = 0.31; I^2^ = 11%) (Figure 3D). Low heterogeneity was noted between the studies (I^2^ = 11%).

#### 3.2.9. LVESD

LVESD was reported by five studies [21,25,26,27,32]. On pooled analysis, patients with sarcoidosis demonstrated a significantly higher LVESD (MD: 0.90; 95% CI: 0.10, 1.71; *p* = 0.03; I^2^ = 41%) compared to healthy controls (Figure 3E). The moderate heterogeneity (I^2^ = 41%) dropped to 0% by omitting Tigen et al., 2015 [32]. However, on omitting Tigen et al., 2015, the pooled estimate shifted towards insignificance (MD: 0.51; 95% CI: −0.16, 1.18; *p* = 0.13; I^2^ = 0%) (Supplementary Figure S9).

#### 3.2.10. E-Wave Velocity

**Figure 4:**
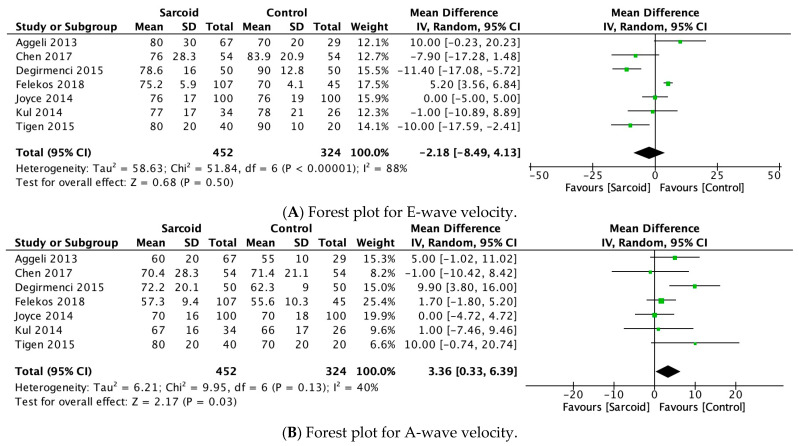

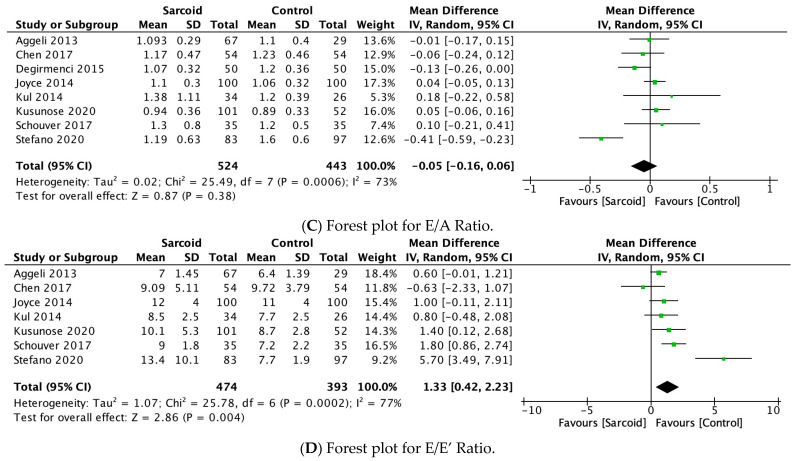
Forest plots comparing echocardiography parameters in patients with sarcoidosis to healthy controls. (**A**) E-wave velocity; (**B**) A-wave velocity; (**C**) E/A ratio; (**D**) E/E’ ratio.

E-wave velocity was reported by seven studies [21,22,24,25,26,27,32]. No significant difference in E-wave velocity was noted between the two groups (MD: −2.18; 95% CI: −8.49, 4.13; *p* = 0.50; I^2^ = 88%) (Figure 4A). High heterogeneity was noted between the studies (I^2^ = 88%), which was reduced to 74% by omitting Felekos et al., 2018 (Supplementary Figure S10) [25].

#### 3.2.11. A-Wave Velocity

A-wave velocity was reported by seven studies [21,22,24,25,26,27,32]. In the pooled analysis, significantly higher A-wave velocity was noted in patients with sarcoidosis (MD: 3.36; 95% CI: 0.33, 6.39; *p* = 0.03; I2 = 40%) compared to healthy controls (Figure 4B). The moderate heterogeneity (I^2^ = 40%) was reduced to 0% by omitting Değirmenci 2015 [24]. However, on omitting Değirmenci 2015 [24], the effect estimate shifted to insignificance (MD: 1.95; 95% CI: −0.36, 4.25; *p* = 0.10; I^2^ = 0%) (Supplementary Figure S11).

#### 3.2.12. E/A Ratio

The E/A ratio was reported by eight studies [17,21,22,24,26,27,28,31]. No significant difference in the E/A ratio was noted between the two groups (MD: −0.05; 95% CI: −0.16, 0.06; *p* = 0.38; I^2^ = 73%) (Figure 4C). The moderate heterogeneity (I^2^ = 73%) was reduced to 12% by omitting Stefano 2020 (Supplementary Figure S12) [17].

#### 3.2.13. E/E’ Ratio

E/E’ ratio was reported by seven studies [17,21,22,26,27,28,31]. In the pooled analysis, patients with sarcoidosis demonstrated a significantly higher E/E’ ratio (MD: 1.33; 95% CI: 0.42, 2.23; *p* = 0.004; I^2^ = 77%) compared to healthy controls (Figure 4D). The high heterogeneity between the studies (I^2^ = 77%) was reduced to 39% by omitting Stefano 2020 (Supplementary Figure S13) [17].

### 3.3. Quality Assessment and Publication Bias

Using the NOS, all studies included in this meta-analysis were deemed to be of “high” methodological quality with scores ≥7 for all studies (Supplementary Table S4). Using funnel plots, no to low asymmetry was visualized for all parameters, hence, a low risk of publication bias was noted (Supplementary Figures S14–S26).

## 4. Discussion

Sarcoidosis is a multisystem granulomatous disorder of unknown etiology, marked by the presence of non-caseating granulomas in affected tissues. Its prevalence is estimated at 10–40 cases per 100,000 individuals in the United States and Europe [33,34,35]. The condition is thought to result from an immune response to an unidentified environmental antigen in genetically predisposed individuals [36]. Antigen presentation through major histocompatibility complex II (MHC-II) activates type 1 T-helper cells, which stimulate the release of various cytokines and chemokines, including IFN-γ, tumor necrosis factor (TNF)-α, transforming growth factor (TGF)-β, IL-2, IL-12, and others. This immune cascade drives granuloma formation, characterized by a core of mononuclear cells encircled by CD4+ lymphocytes, along with smaller populations of CD8+ and B cells [37,38]. Granulomatous inflammation can progress to fibrosis, leading to irreversible organ damage over time. In pulmonary sarcoidosis, fibrosis accumulates gradually, often resulting in respiratory failure after more than a decade. By contrast, CS involves granulomas within the heart and is typically more acute and potentially life-threatening. Although many cases are asymptomatic or subclinical, symptomatic CS can cause HF, heart block, or severe arrhythmias. Sudden death from CS is a major concern due to its acute presentation and poor prognosis, highlighting the critical importance of early diagnosis and intervention [3,39].

This systematic review and meta-analyses represent one of the most comprehensive evaluations to date, incorporating the largest cohort of retrospective studies and patient populations to assess echocardiography-based cardiovascular function parameters in individuals with sarcoidosis compared to healthy controls. A total of thirteen studies were included, comprising 854 sarcoidosis patients and 526 controls, resulting in a combined sample size of 1407 participants. The pooled analysis revealed that sarcoidosis is significantly associated with a reduced LV GLS, LV GCS, and a notable increase in A-wave velocity and the E/E’ ratio. However, no significant changes were observed in LVGRS, TAPSE, PASP, IVSD, E-wave velocity, E/A ratio, and LVEDD between the two groups.

First, our analysis identified a significant reduction in LV GLS in patients with sarcoidosis, highlighting its role as a sensitive and prognostic marker for subclinical myocardial involvement. Decreased GLS values are associated with an increased risk of adverse outcomes, including cardiovascular death, cardiac dysfunction, high-grade atrioventricular block, and malignant ventricular arrhythmias [26]. In support of this, a study by Cameli et al. found significantly lower LV GLS in sarcoidosis patients who experienced major adverse cardiovascular events (MACE) (*p* = 0.025). An LV GLS value of <17.13% (absolute value) was identified as a reliable predictor of MACE, further emphasizing its prognostic value [40]. Similarly, Murtagh et al. reported a GLS cutoff of −17%, demonstrating a sensitivity and specificity of 94% for detecting CS. Additionally, patients who experienced major cardiac events (MCEs) had significantly worse GLS compared to those who did not (−13.4 ± 0.9% vs. −17.7 ± 0.4%, *p* = 0.0003) [41].

The observed reduction in LV GLS in sarcoidosis is primarily attributed to the preferential localization of inflammatory granulomas in the mid-myocardial layer of the left ventricular wall, which is responsible for longitudinal deformation. These granulomas induce fibrotic changes and scar formation, leading to impaired LV mechanics. Early myocardial involvement in sarcoidosis is typically patchy and localized, causing reduced LV GLS as the first sign of contractile dysfunction, even before a decrease in LVEF is observed [42]. Furthermore, myocardial deformation imaging proves valuable for detecting early dysfunction in CS patients [43]. The LV wall consists of three layers: subendocardial, mid-wall, and subepicardial, with the early impairment of GLS reflecting disruption of the longitudinally organized myofibrils, predominantly located in the subendocardial layer. While CS commonly affects the epicardial and mid-wall regions, as evidenced by late gadolinium enhancement-CMR (LGE-CMR) imaging, GLS is often impaired at the early stages of the disease [44]. In accordance with these findings, Kansal et al. studied patients with various cardiomyopathies, including CS, and found a significant reduction in GLS, regardless of the distribution of LGE within myocardial layers [44]. These findings suggest that functional decline in CS may exceed the structural damage seen on imaging, reinforcing the value of strain parameters, such as GLS for early detection and prognostic evaluation.

Second, our results revealed a significant reduction in LV GCS in patients with sarcoidosis, aligning with the findings of Tigen et al., Kul et al., and Orii et al., who also observed a notable decrease in GCS compared to healthy controls [27,29,32]. In patients with CS, LV GCS is generally lower than in healthy individuals, though findings across studies have been inconsistent. Kull et al. reported a significantly reduced LV GCS in sarcoidosis patients (−17.7% ± 4.9%) compared to healthy controls (−22.7% ± 3.1%, *p* < 0.001), reinforcing its potential role in identifying myocardial dysfunction. Similarly, Orii et al. demonstrated that segments with late gadolinium enhancement (LGE) had a markedly lower peak circumferential strain (−14% ± 6%) than both non-LGE segments (−28% ± 7%) and control segments (−30% ± 7%) (*p* < 0.0001), highlighting the association between LV GCS reduction and myocardial fibrosis [27,29,30]. However, both Di Stefano et al. and Schouver et al. found no significant difference in LV GCS between sarcoidosis patients and healthy controls, suggesting variability in findings across studies [17,31]. This inconsistency may be attributed to differences in sample size and study power, particularly for LV GCS, where group differences may be less pronounced. While LV GCS appears to be reduced in cardiac sarcoidosis, no specific prognostic cutoff values have been established, limiting its clinical applicability in risk stratification.

Circumferential strain is derived from the shortening of fibers along the circular perimeter of the myocardium. In CS, the presence of myocardial fibrosis—often affecting the mid-wall as compared to the epicardial and endocardial wall—leads to the disruption of normal myocardial architecture, impairing the heart’s contractile function, especially in the circumferential direction, which is mainly a result of granulomatous inflammation [45,46,47]. Consequently, mid-wall fibrosis is commonly linked to reduced GCS in non-ischemic cardiomyopathies. Impaired segmental circumferential strain values measured by STE have been shown to correlate with myocardial damage detected on CMR imaging [29]. While some studies have demonstrated a significant reduction in LV GCS in CS, the role of LV-GCS in diagnosing and screening for this condition remains unclear. While some research indicates substantial reductions in LV-GLS, others, including those by De Stefano et al. and Schouver et al., found no significant difference between healthy controls and patients with sarcoidosis. This variability highlights the need for further prospective studies using robust diagnostic criteria to better understand the role of both LV-GCS and LV-GLS in the diagnosis and screening of CS.

Third, our analysis revealed a notable increase in A-wave velocity and the E/E’ ratio, reflecting diastolic dysfunction and myocardial involvement in sarcoidosis. The elevated A-wave velocity suggests a compensatory mechanism during early diastolic dysfunction, where the left atrium generates stronger contractions to maintain adequate ventricular filling despite impaired relaxation [48]. These findings are consistent with the pathological features of CS, such as myocardial granulomas and fibrosis, which compromise left ventricular compliance [3,24]. The elevated E/E’ ratio serves as an indicator of increased LV filling pressures, signaling advanced diastolic dysfunction in sarcoidosis [49]. The E/E’ ratio of 13.4 reported by Stefano et al. was the mean value observed in patients with cardiac sarcoidosis, compared to the normal reference value of <8, indicating abnormal LV filling pressures [17]. Ozyilmaz et al. reported a significantly higher prevalence of diastolic dysfunction in both the LV and RV in CS patients compared to healthy controls. This aligns with previous findings demonstrating that an increased E/E’ ratio may potentially be associated with worsening diastolic function, further highlighting its potential role in assessing disease severity in cardiac sarcoidosis [50]. However, traditional Doppler parameters, such as E, A, and E/A ratios, are preload- and afterload-dependent and may not accurately reflect diastolic function in patients with preserved LVEF. Additionally, the diagnostic accuracy of E/E’ in estimating LV filling pressures in preserved EF is limited, as highlighted by Sharifov et al., who found insufficient evidence to support its reliability in this context [51]. Diastolic dysfunction is an independent predictor of all-cause mortality, even in its preclinical stage, and is essential for diagnosing HFpEF. Consequently, the accurate assessment of LV diastolic dysfunction is critical in clinical practice. Pritchett et al. demonstrated in a cohort of 2042 participants that LA volume indexed to body surface area (LAVi) increased progressively with worsening diastolic dysfunction: 23 ± 6 mL/m^2^ in normal diastolic function, 25 ± 8 mL/m^2^ in grade I diastolic dysfunction, 31 ± 8 mL/m^2^ in grade II diastolic dysfunction, and 48 ± 12 mL/m^2^ in grades III to IV diastolic dysfunction [52].

Furthermore, our results align with the study by Tigen et al., who also demonstrated significantly lower LA GLS (left atrium global longitudinal strain) values in sarcoidosis patients compared to healthy controls (34.3 ± 3.6% vs. 39.1 ± 4.1%, *p* = 0.001) [32]. Additionally, atrial wall thickening and left ventricular impairment have been previously reported in patients with CS. These observations highlight the importance of detailed imaging of the left atrium in sarcoidosis patients, particularly those presenting with atrial arrhythmias, as this may enhance the sensitivity of echocardiographic diagnosis for CS [53,54]. The elevated E/E’ ratio serves as an indicator of increased LV filling pressures, signaling advanced diastolic dysfunction in sarcoidosis [49]. Ozyilmaz et al. reported a significantly higher prevalence of diastolic dysfunction in both the LV and RV in CS patients compared to healthy controls [50]. Additionally, a systematic review and meta-analysis by Okasha et al. identified frequent sites of myocardial involvement, including LV subepicardial, septal, and RV-free wall regions, corresponding to granulomatous inflammation and fibrosis [55]. Miyakuni et al. further emphasized the elevated NT-proBNP levels in CS, a marker of ventricular stress linked to adverse cardiovascular outcomes and the progression toward HF [56]. These pathological changes may contribute to HF with preserved ejection fraction (HFpEF) or HF with reduced ejection fraction (HFrEF) and, in severe cases, restrictive cardiomyopathy [57]. Furthermore, RV dysfunction may arise due to direct granulomatous infiltration or secondary effects from LV failure or pulmonary hypertension, worsening clinical outcomes [26]. Our analysis revealed a significant increase in LVESD in patients with sarcoidosis, indicative of a deteriorating myocardial function [58]. This phenomenon is likely due to the impaired contractility of the left ventricle resulting from granulomatous inflammation and fibrosis in sarcoidosis, thereby resulting in progressive LV insufficiency [58,59]. These structural changes in the LV contribute to the development of systolic dysfunction and may progress to HF, thereby highlighting the role of ventricular remodeling in the pathophysiology of CS.

Lastly, our analysis revealed significantly increased PASP in patients with CS, which may be elevated due to several mechanisms. Sarcoidosis-associated pulmonary hypertension (SAPH) can result from granulomatous inflammation directly involving the pulmonary vasculature, leading to vascular remodeling and increased resistance [60]. Additionally, fibrotic changes within the lung parenchyma can cause hypoxic vasoconstriction, further elevating PASP [61]. According to a meta-analysis conducted by Zhang et al., the pooled prevalence of SAPH was 16.4% by transthoracic echocardiography and 6.4% by right heart catheterization (RHC) in the general sarcoidosis population. In advanced sarcoidosis, SAPH prevalence increased significantly to 62.3% by RHC, with precapillary pulmonary hypertension being the predominant type [62]. Overall, the burden of pulmonary hypertension (PH) in sarcoidosis remains relatively low but rises substantially with disease progression [62]. The extrinsic compression of pulmonary arteries by enlarged lymph nodes or sarcoid granulomas may also contribute to increased pulmonary pressures [63]. Moreover, left ventricular diastolic dysfunction, common in CS, can lead to elevated left heart pressures that transmit backward into the pulmonary circulation, raising PASP [63]. These factors collectively contribute to the observed increase in PASP among CS patients.

Our analysis revealed no significant differences in LVEF between the two groups. This preservation of systolic function is characteristic of early or patchy CS, where the extent of myocardial damage is insufficient to impair global function. However, as granulomatous inflammation and fibrosis progress, leading to significant myocardial involvement, LVEF eventually declines [64,65]. This may be due to the presence of early or subclinical CS in the patient population analyzed, where damage was not yet severe enough to affect systolic function. Similarly, LVGRS was unaffected, reflecting the potential sparing of regions critical for radial strain due to the patchy distribution of fibrosis [66]. TAPSE, a marker of RV function, also showed no significant difference, as RV involvement is less frequent in CS (reported in 36–39% of autopsy studies) and typically occurs later in disease progression [67,68]. PASP was also comparable between the two groups, given that isolated CS rarely leads to pulmonary hypertension unless coexisting with severe pulmonary sarcoidosis [63]. No significant changes were observed in IVSD, as the localized involvement of myocardial sarcoidosis does not typically result in global septal thickening, since the LV walls are the most affected site [69]. Similarly, E-wave velocity and the E/A ratio, markers of diastolic function, remained unaffected, likely due to intact compensatory mechanisms or milder disease in the studied population [70]. Lastly, LVEDD showed no significant enlargement, reflecting minimal ventricular remodeling in the absence of advanced or extensive myocardial damage [71,72].

With regard to effective diagnostic modalities, TTE is a primary screening tool for CS, revealing abnormalities like regional wall motion defects and myocardial echogenicity [63,73]. While TTE findings suggest potential cardiac involvement, further diagnostic tests, such as PET or CMR, are essential for confirmation as per the Heart Rhythm Society consensus [69]. Significant basal septal thinning and reduced LVEF, markers of advanced CS, are linked to worse outcomes [74,75]. Additionally, GLS is a more sensitive and prognostic marker for early myocardial involvement in CS compared to GCS and GRS. It measures the longitudinal deformation of subendocardial fibers, which are the first to be affected due to their higher susceptibility to ischemia and fibrosis. This allows for the early detection of dysfunction even in patients with preserved LVEF [42,43,44]. A cohort study by Stefano et al. found a significant association between GLS reduction and an increased risk of hospital admission and progression to HF in CS patients with preserved LVEF [17]. In contrast, GCS reflects mid-wall fiber function, but its decline in CS remains inconsistent across studies. GRS, which assesses radial thickening, is less reliable due to greater variability and later-stage involvement [17,27,29,30,31]. Studies have demonstrated that reduced GLS is strongly associated with an increased risk of MACE, including malignant arrhythmias and AV block, reinforcing its prognostic value [40,41]. Given its superior sensitivity and strong correlation with adverse outcomes, GLS remains the most reliable strain parameter for early detection and risk stratification in CS.

### Limitations

Some limitations must be considered when interpreting the results of this meta-analysis. First, the inclusion of observational studies introduces residual confounders and heterogeneity in echocardiography parameters. Observational studies are at high risk of selection and reporting bias. Second, the sample size is small; further large-scale multicentric prospective studies are warranted. Third, this is a study-level meta-analysis, and no individual-patient data analysis could be conducted. This leads to potential case-to-case variations which could not be accounted for. Fourth, the baseline variability in population cohorts introduced high heterogeneity for multiple parameters in this meta-analysis. Fifth, the co-existence of hypertension, diabetes mellitus (DM), smoking, and dyslipidemia (in some studies) may independently alter echocardiographic parameters, irrespective of the presence of subclinical CS, further contributing to variability in the findings. Finally, the absence of reported clinical outcomes in the included studies limits our ability to assess the prognostic value of strain imaging in predicting cardiovascular complications. Future studies should incorporate long-term follow-up data to evaluate its utility in risk stratification and clinical decision-making.

## 5. Conclusions

In conclusion, CS is a complex condition with significant cardiovascular implications. Our meta-analysis highlights the importance of echocardiographic parameters, such as GLS and GCS, as early indicators of myocardial involvement. These findings highlight the potential of imaging techniques for diagnosing and monitoring CS and guiding clinical management. Given its patchy and often subclinical nature, early detection is essential to prevent irreversible damage and improve outcomes, emphasizing the need for comprehensive imaging and timely intervention in high-risk individuals.

## Figures and Tables

**Table 1:** Baseline characteristics of included studies.

Study	Country	Patient Population	STE Software	Number of Participants		Females (*N*)		Mean Age		Organ Involvement (%)	Hypertension (%)		Diabetes (%)		Smoking (%)		Dyslipidemia (%)		History of Steroid Usage (%)
				S	C	S	C	S	C		S	C	S	C	S	C	S	C	
Aggeli 2023 [21]	Greece	Newly diagnosed sarcoidosis patients	QLAB 9.0	67	29	41	18	46 ± 2.6	45.2 ± 1.9	Lung—91.8% Skin—18% Eye—6.5%	0		0		0		0		0
Chen 2017 [22]	United States	Sarcoidosis patients with no proven cardiac involvement	NR	54	54	22	18	51.3 ± 11.2	51 ± 10.4	Lung—96.3% Neuro—5.6% Liver—1.9% Eye—1.9% Osseous—1.9% Skin—3.7%	59.3	59.3	37	29.6	64.8	40.7	38.9	24.1	33.3
Dabir 2018 [23]	Germany	Biopsy-proven sarcoidosis	View Forum, Philips Healthcare	38	22	18	22	52.7 ± 12	59.3 ± 17	Lung—92% Lymph node—8% Liver—11% Skin—11% Spleen—5% Bone—5% Eye—11%	16	0	8	0	13	0	11	0	63
Degirmenci 2015 [24]	Turkey	Recently diagnosed sarcoidosis patients	Echo-Pac Version 7.0, GE Vingmed	50	50	34	10	40.4 ± 11	37.7 ± 4.9	Pulmonary—100%	0		0		56	60	0		0
Felekos 2018 [25]	Greece	Extracardiac sarcoidosis patients	QLAB 9.0	107	45	7	26	47 ± 14	45 ± 8	Pulmonary—88.8% Skin—18.7% Eye—7.5%	5.6	2.2	0	2.2	NR		NR		NR
Joyce 2015 [26]	Netherlands	Biopsy-proven sarcoidosis patients attending a referral clinic	EchoPac 112.0.1	100	100	52	32	55 ± 13	55 ± 13	Lung—90% Skin—36% Joint—27% Eye—17% Renal—12% Neurological—8% Liver—6% Parotid—4% Other—4%	24	27	10	8	26	36	26	21	18
Kul 2014 [27]	Turkey	Biopsy-proven pulmonary sarcoidosis of grade 1 or 2	QLab 9.0, Philips	34	26	25	52	45 ± 9.1	44.3 ± 9	Lung—100% Skin—15% Eye—2.5% Lymph nodes—17.5%	0		0		NR		0		NR
Kusunose 2020 [28]	Japan	Biopsy-proven sarcoidosis patients	EchoInsight, Epsilon Imaging	101	52	69	18	62 ± 13	62 ± 11	Lung—63% Eye—41% Skin—25% Nerve—5% Muscle—4% Kidney—2% Liver—2% Stomach—1% Lymph node—1%	35	37	40	38	NR		NR		NR
Orii 2015 [29]	Japan	Biopsy-proven extracardiac sarcoidosis	EchoPAC, GE Vingmed	32	10	20	34	57 ± 13	57 ± 13	Lung—72%	16	0	3	0	NR		16	0	16
Panovsky 2021 [30]	Czech Republic	Sarcoidosis of the respiratory tract and/or extrapulmonary sarcoidosis	Image-Arena Version 4.6.4.40	113	22	51	6	52 ± 10.8	52.7 ± 11.1	Lung—100% Extrapulmonary—30%	31	31.8	5.3	0	NR		NR		56
Schouver 2017 [31]	France	Biopsy-proven sarcoidosis	TomTec 2D Cardiac Performance Analysis	35	35	22	22	47.9 ± 14.8	47.5 ± 16.3	Lymph nodes—80% Lung—57.1% Ocular—31.4% Skin—28.6% Articular—17.1% Renal—8.6% Neurological—2.9% Splenic—8.6% Liver—5.6%	14.3	14.3	0	0	22.9	25.7	NR		62.9
Stefano 2020 [17]	United States	Systemic sarcoidosis patients	Image-Arena V4.6 software	83	97	29	61	53.6 ± 10.8	40.4 ± 13.6	Lung—67.5% Lymph node—68.7% Eye—9.6% Skin—12% Neurological—5%	37.3	0	19.3	0	NR		30	0	68.7
Tigen 2015 [32]	Turkey	Biopsy-proven sarcoidosis	EchoPAC 6.1, GE Vingmed	40	20	34	17	46.4 ± 10.5	41.9 ± 12.4		0		0		NR		0		NR

Abbreviations: QLAB, quantification lab software; NR, not reported.

## Supplementary Materials

The following supporting information can be downloaded at: https://www.mdpi.com/article/10.3390/diagnostics15060708/s1, Table S1. The Preferred Reporting Items for Systematic Reviews and Meta-Analyses (PRISMA) 2020 Checklist. Table S2. Search strategy for all databases. Table S3: Inclusion and exclusion criteria of each study. Table S4: Quality Assessment for the included studies using the Newcastle-Ottawa Scale (NOS). Figure S1. Sensitivity analysis for LV GLS. Figure S2. Sensitivity analysis for LV GCS. Figure S3. Sensitivity analysis for LVEF. Figure S4. Sensitivity analysis for LV GRS. Figure S5. Sensitivity analysis for IVST. Figure S6. Sensitivity analysis for TAPSE (omitting Joyce 2015 [26]). Figure S7. Sensitivity analysis for TAPSE (omitting Kusunose 2020 [28]). Figure S8. Sensitivity analysis for PASP. Figure S9. Sensitivity analysis for LVESD. Figure S10. Sensitivity analysis for E-wave velocity. Figure S11. Sensitivity analysis for A-wave velocity. Figure S12. Sensitivity analysis for E/A ratio. Figure S13. Sensitivity analysis for E/E’ ratio. Figure S14. Funnel plot for LV GLS. Figure S15. Funnel plot for LV GCS. Figure S16. Funnel plot for LVEF. Figure S17. Funnel plot for LV GRS. Figure S18. Funnel plot for IVST. Figure S19. Funnel plot for TAPSE. Figure S20. Funnel plot for PASP. Figure S21. Funnel plot for LVEDD. Figure S22. Funnel plot for LVESD. Figure S23. Funnel plot for E-wave velocity. Figure S24. Funnel plot for A-wave velocity. Figure S25. Funnel plot for E/A ratio. Figure S26. Funnel plot for E/E’ ratio.

## Abbreviations

CI	confidence interval
CMR	cardiac magnetic resonance
CS	cardiac sarcoidosis
EMB	endomyocardial biopsy
GCS	global circumferential strain
GLS	global longitudinal strain
GRS	global radial strain
HF	heart failure
IVST	interventricular septal thickness
LGE	late gadolinium enhancement
LV	left ventricular
LVEDD	left ventricular end-diastolic diameter
LVEF	left ventricular ejection fraction
LVESD	left ventricular end-systolic diameter
MACE	major adverse cardiovascular events
MD	mean difference
MHC	major histocompatibility complex
NOS	Newcastle–Ottawa scale
PASP	pulmonary artery systolic pressure
PET	positron emission tomography
RV	right ventricular
SCD	sudden cardiac death
STE	speckle tracking echocardiography
TAPSE	tricuspid annular plane systolic excursion
TGF	transforming growth factor
TNF	tumor necrosis factor
TTE	transthoracic echocardiography
