# Supplementary material for: Diagnostic Value of Comprehensive Echocardiographic Assessment Including Speckle-Tracking in Patients with Sarcoidosis Versus Healthy Controls: A Systematic Review and Meta-Analysis
Source: Diagnostics (Basel). 2025 Mar 12;15(6):708. doi: 10.3390/diagnostics15060708 (PMC11941600; doi:10.3390/diagnostics15060708)

Supplementary Material

Diagnostic Value of Speckle-Tracking Echocardiography Parameters in Patients with Sarcoidosis Versus Healthy Controls: A Systematic Review and Meta-Analysis

Supplementary Table S1. The Preferred Reporting Items for Systematic Reviews and Meta-Analyses (PRISMA) 2020 Checklist.

Section and Topic	Item #	Checklist item	Location where item is reported
**TITLE**			
Title	1	Identify the report as a systematic review.	1
**ABSTRACT**			
Abstract	2	See the PRISMA 2020 for Abstracts checklist.	1, 2
**INTRODUCTION**			
Rationale	3	Describe the rationale for the review in the context of existing knowledge.	5, 6
Objectives	4	Provide an explicit statement of the objective(s) or question(s) the review addresses.	5, 6
**METHODS**			
Eligibility criteria	5	Specify the inclusion and exclusion criteria for the review and how studies were grouped for the syntheses.	7
Information sources	6	Specify all databases, registers, websites, organisations, reference lists and other sources searched or consulted to identify studies. Specify the date when each source was last searched or consulted.	7
Search strategy	7	Present the full search strategies for all databases, registers, and websites, including any filters and limits used.	7
Selection process	8	Specify the methods used to decide whether a study met the inclusion criteria of the review, including how many reviewers screened each record and each report retrieved, whether they worked independently, and if applicable, details of automation tools used in the process.	7, 8, 9
Data collection process	9	Specify the methods used to collect data from reports, including how many reviewers collected data from each report, whether they worked independently, any processes for obtaining or confirming data from study investigators, and if applicable, details of automation tools used in the process.	7, 8, 9
Data items	10a	List and define all outcomes for which data were sought. Specify whether all results that were compatible with each outcome domain in each study were sought (e.g. for all measures, time points, analyses), and if not, the methods used to decide which results to collect.	8, 9
	10b	List and define all other variables for which data were sought (e.g. participant and intervention characteristics, funding sources). Describe any assumptions made about any missing or unclear information.	8, 9
Study risk of bias assessment	11	Specify the methods used to assess risk of bias in the included studies, including details of the tool(s) used, how many reviewers assessed each study and whether they worked independently, and if applicable, details of automation tools used in the process.	9

Effect measures	12	Specify for each outcome the effect measure(s) (e.g. risk ratio, mean difference) used in the synthesis or presentation of results.	
Synthesis methods	13a	Describe the processes used to decide which studies were eligible for each synthesis (e.g. tabulating the study intervention characteristics and comparing against the planned groups for each synthesis (item #5)).	9
	13b	Describe any methods required to prepare the data for presentation or synthesis, such as handling of missing summary statistics, or data conversions.	9
	13c	Describe any methods used to tabulate or visually display results of individual studies and syntheses.	9
	13d	Describe any methods used to synthesize results and provide a rationale for the choice(s). If meta-analysis was performed, describe the model(s), method(s) to identify the presence and extent of statistical heterogeneity, and software package(s) used.	9
	13e	Describe any methods used to explore possible causes of heterogeneity among study results (e.g. subgroup analysis, meta-regression).	9
	13f	Describe any sensitivity analyses conducted to assess robustness of the synthesized results.	9
Reporting bias assessment	14	Describe any methods used to assess risk of bias due to missing results in a synthesis (arising from reporting biases).	NA
Certainty assessment	15	Describe any methods used to assess certainty (or confidence) in the body of evidence for an outcome.	NA
**RESULTS**			
Study selection	16a	Describe the results of the search and selection process, from the number of records identified in the search to the number of studies included in the review, ideally using a flow diagram.	10
	16b	Cite studies that might appear to meet the inclusion criteria, but which were excluded, and explain why they were excluded.	10
Study characteristics	17	Cite each included study and present its characteristics.	10
Risk of bias in studies	18	Present assessments of risk of bias for each included study.	14
Results of individual studies	19	For all outcomes, present, for each study: (a) summary statistics for each group (where appropriate) and (b) an effect estimate and its precision (e.g. confidence/credible interval), ideally using structured tables or plots.	10, 11, 12, 13, 14
Results of syntheses	20a	For each synthesis, briefly summarise the characteristics and risk of bias among contributing studies.	10, 11, 12, 13, 14
	20b	Present results of all statistical syntheses conducted. If meta-analysis was done, present for each the summary estimate and its precision (e.g. confidence/credible interval) and measures of statistical heterogeneity. If comparing groups, describe the direction of the effect.	10, 11, 12, 13, 14
	20c	Present results of all investigations of possible causes of heterogeneity among study results.	10, 11, 12, 13, 14

	20d	Present results of all sensitivity analyses conducted to assess the robustness of the synthesized results.	10, 11, 12, 13, 14
Reporting biases	21	Present assessments of risk of bias due to missing results (arising from reporting biases) for each synthesis assessed.	NA
Certainty of evidence	22	Present assessments of certainty (or confidence) in the body of evidence for each outcome assessed.	NA
**DISCUSSION**			
Discussion	23a	Provide a general interpretation of the results in the context of other evidence.	15, 16, 17, 18, 19
	23b	Discuss any limitations of the evidence included in the review.	20
	23c	Discuss any limitations of the review processes used.	20
	23d	Discuss implications of the results for practice, policy, and future research.	18, 19
**OTHER INFORMATION**			
Registration and protocol	24a	Provide registration information for the review, including register name and registration number, or state that the review was not registered.	7
	24b	Indicate where the review protocol can be accessed, or state that a protocol was not prepared.	7
	24c	Describe and explain any amendments to information provided at registration or in the protocol.	7
Support	25	Describe sources of financial or non-financial support for the review, and the role of the funders or sponsors in the review.	Title Page
Competing interests	26	Declare any competing interests of review authors.	Title Page
Availability of data, code and other materials	27	Report which of the following are publicly available and where they can be found: template data collection forms; data extracted from included studies; data used for all analyses; analytic code; any other materials used in the review.	Title Page

Supplementary Table S2. Search strategy for all databases.

No.	Database	Search Strategy	Number of Articles
1.	PubMed	(Sarcoidosis) AND ((Echocardiography) OR (Myocardial deformation) OR (Speckle Tracking) OR (Doppler Echocardiography))	645
2.	Embase	(Sarcoidosis) AND ((Echocardiography) OR (Myocardial deformation) OR (Speckle Tracking) OR (Doppler Echocardiography))	512
3.	Web of Science	(TS= (“sarcoidosis”)) AND (TS= (“echocardiography”) OR TS= (“myocardial deformation”) OR TS= (“doppler echocardiography”) OR TS= (“speckle tracking”))	348
4.	Scopus	((TITLE-ABS-KEY (sarcoidosis)) AND (TITLE-ABS-KEY (echocardiography) OR TITLE-ABS-KEY (myocardial deformation) OR TITLE-ABS-KEY (doppler echocardiography) OR TITLE-ABS-KEY (speckle tracking)))	395
5.	Cochrane	(MeSH descriptor: [Sarcoidosis] explode all trees) AND ((MeSH descriptor: [echocardiography] explode all trees)) AND ((MeSH descriptor: [Myocardial deformation] explode all trees) OR (MeSH descriptor: [Speckle tracking] explode all trees))	68
TOTAL			1968

Supplementary Table S3: Inclusion and exclusion criteria of each study.

Study	Inclusion criteria	Exclusion criteria
Aggeli 2023	Seventy-six patients with newly diagnosed sarcoidosis were referred to our tertiary center for echocardiographic evaluation. All patients had biopsy-proven disease. In addition, no patient was under treatment at the time of the examination, as this could have acted as a confounding factor for our results.	Exclusion criteria included the following: any symptoms and signs of heart disease, the administration of any medication affecting the cardiovascular system, smoking, the presence of diabetes mellitus, dyslipidemia or hypertension, known structural or valvular heart disease, as well as poor acoustic window.
Chen 2017	Extracardiac sarcoidosis patients with cardiac symptoms (chest pain, dyspnea, palpitation, or syncope) or abnormal ECG changes in our sarcoidosis clinic from December 2010 to June 2015. Sarcoidosis diagnosis was confirmed in all cases by both clinical data and histological evidence of non-caseating granuloma. Either invasive (cardiac catheterization) or noninvasive (echo cardiac or nuclear stress test) workup has been performed in all studied patients to rule out CAD.<br><br>Fifty-four non-sarcoidosis patients were included as controls. They were initially referred for echocardiographic studies for chest pain, dyspnea, palpitation, or syncope, or abnormal ECG changes, in the absence of any prior history of structural heart disease. An age- and gender-matched comparator group was selected in a 1:1 ratio.	Patients with a previous diagnosis of CS (n= 20) or other structural heart diseases (n=10) were excluded.
Dabir 2018	The study population consisted of outpatients receiving treatment at the local department of pneumology and patients recruited from a nationwide sarcoidosis support group (Sarkoidose-Netzwerk e. V.).	Except for patients with a history of arterial hypertension, none of the patients included in this study had a history of significant cardiac disease (e. g. myocardial infarction, myocarditis, cardiomyopathy)
Degirmenci 2015	All patients were referred by the outpatient department of the pulmonology clinic of the medical faculty. All the patients had biopsy-proven disease identified through mediastinoscopy, thoracoscopy or bronchoscopy. None of the patients had cardiac symptoms or ECG findings related to CS.	Exclusion criteria included coronary artery disease, hypertension, diabetes mellitus, renal failure, chronic obstructive pulmonary disease, heart failure, systolic LV dysfunction, moderate or severe valvular heart disease, AF, thyroid dysfunction or parathyroid dysfunction, and connective tissue disease. Arrhythmias were excluded by Holter electrocardiography (ECG).
Felekos 2018	One hundred seventeen patients with extracardiac sarcoidosis who were referred to our tertiary center for echocardiographic assessment were retrospectively enrolled in this study. The diagnosis of sarcoidosis required a compatible clinical picture with or without histological evidence of noncaseating granulomas and the absence of another disease process capable of producing a similar picture. Prior referrals to our echo laboratory, patients were assessed clinically for any signs of cardiovascular involvement by an independent cardiologist.<br><br>45 age-and sex- matched healthy controls were included in the study.	Patients with symptoms indicative of cardiac involvement (defined as palpitations, presyncope, syncope, non-pleuritic chest pain, and dyspnea related to congestive heart failure), abnormal ECG, previously known structural disease or established CS based on the Japanese Ministry for Health and Welfare (JMWH) diagnostic criteria were excluded from our study.
Joyce 2014	One-hundred and thirty consecutive patients with sarcoidosis attending our tertiary referral centre were identified from the departmental Cardiology Information System (EPD-Vision®, Leiden University Medical Center). Patients were required to have undergone 2D-TTE around the time of, or following, their diagnosis.<br><br>Consecutive controls were identified from the departmental echocardiographic database (EchoPac) using a specific echocardiographic search code identifying the absence of any structural cardiac abnormalities. Initial referral for echocardiography included chest pain, dyspnoea, syncope, palpitations, murmur on auscultation, or cardiovascular risk stratification, in the absence of any prior history of structural heart disease. The absence of structural cardiac disease was defined as a 'normal' echocardiogram according to American Society of Echocardiography guidelines.<br><br>An age- and gender-matched comparator group was selected in a 1:1 ratio according to published comparability principles, in that they were representative of the same base experience as cases, but without any prior or current history of sarcoidosis.	Patients were excluded if they had known structural heart disease (n = 16) or definite CS (n = 5) at the time of the first 2DTTE study (date of study enrolment). Regarding those excluded for known structural heart disease, the majority (n = 10) had significant ischaemic heart disease, as defined by >50% stenosis in >/=1 epicardial vessel and/or prior acute coronary syndrome and/or prior revascularization procedure, three due to congenital heart disease, two due to known dilated cardiomyopathy from another cause, and one due to significant valve disease, defined as >/= 2 regurgitation or moderate or more stenosis. The presence of CS was defined by fulfilment of the histological or clinical diagnosis group criteria of the revised JMHW guidelines.11 Those who fulfilled one major criterion of the updated guidelines at study enrolment (n = 9) were also excluded, as this was considered to indicate high likelihood of future CS diagnosis.
Kul 2014	Forty patients with biopsy-proven pulmonary sarcoidosis of grade 1 or 2 and 26 healthy subjects matched by age, body mass index, heart rate, and blood pressure, were prospectively enrolled. The diagnosis of sarcoidosis was histopathologically confirmed according to the consensus criteria of the American Thoracic Society (ATS)/European Respiratory Society (ERS).	Patients with known HT, diabetes mellitus (DM), HF, atrial fibrillation (AF), coronary artery disease (CAD), more than a mild valvular disorder, an implanted pacemaker/defibrillator, and/or thyroid, liver, and renal disease were excluded.
Kusunose 2020	All sarcoidosis patients without cardiac involvement had radionuclide scanning (n = 69), cardiac magnetic resonance imaging (n = 18), or both advanced imaging tests (n = 14) at baseline.<br><br>Fifty-two age- and gender-matched control patients were selected from our volunteer database comprised of comprehensive histories and physical examinations. All examinations (laboratory data, echocardiographic examinations, and advanced cardiovascular imaging) have done within a month.	Excluded patients with other pre-existing structural heart diseases and poor echocardiographic images.
Orii 2015	Forty-eight patients who presented with biopsy-proven extracardiac sarcoidosis were retrospectively screened between May 2012 and December 2013 at Wakayama Medical University Hospital.<br><br>Ten age-matched, completely healthy control subjects were recruited as a control group, with no evidence of sarcoidosis, coronary artery disease, or valvular disease; these subjects did not have noncardiac comorbidities such as hepatic, renal, and malignant diseases. All subjects underwent conventional and color Doppler echocardiography and DE MRI on the same day.	Exclusion criteria were clinical findings of cardiac involve-ment, including LV dysfunction (LV ejection fraction [LVEF] < 50%) and abnormal electrocardiographic (atrioventricular block, ventricular arrhythmias, complete right and left bundle branch block, axis deviation, and abnormal Q wave) and echocardiographic (wall thinning, regional abnormal wall motion, and ventricular aneurysm) findings according to Japanese Ministry of Health and Welfare criteria. Other exclusion criteria included atrial fibrillation. Among the 48 patients, one was excluded for an LVEF < 50%, one for right bundle branch block, and one for atrioventricular nodal conduction disease. No patients were excluded because of contraindications to MRI (severe renal impairment, implantable cardioverter-defibrillator, or pacemaker).
Panovsky 2021	Patients with sarcoidosis of the respiratory tract and/or extrapulmonary sarcoidosis and available CMR examination were enrolled into this retrospective study	Patients with Löfgren syndrome were excluded. Patients with any pre-existing known cardiovascular disease or cardiac symptoms or any signs of cardiac involvement of sarcoidosis prior to screening were also excluded.
Schouver 2017	Patients were recruited in the University Hospital of Nice respiratory and internal medicine departments. We included consecutive adult patients with a diagnosis of sarcoidosis based on the American thoracic society (ATS)/European respiratory society (ERS)/World association of sarcoidosis and other granulomatous disorders (WASOG) criteria.<br><br>An age- and gender-matched healthy control population was enrolled in a 1:1 ratio. Control patients were also"blood pressure" matched by degree of systolic blood pressure: 100-120 mm Hg or 120–140 mm Hg. Consecutive controls were identified from the echocardiography department in the absence of any structural cardiac abnormalities. Initial referral causes for echocardiography	Patients with suspected or confirmed CS according to the HRS 2014 expert consensus statement [10] and those with known or suspected structural heart disease were excluded from the study. Sarcoidosis patients were included at the time of their normal TTE.<br><br>Controls had no history of structural heart disease or sarcoidosis and were included at the time of their normal TTE.

	included palpitations, dyspnoea, chest pain and cardiovascular risk factors assessment. A “normal” ultrasound was defined according to the latest European Association for Cardiovascular Imaging (EACVI)/ American society of Echocardiography (ASE) guidelines.	
Stefano 2020	We retrospectively identified 122 patients with systemic sarcoidosis that were evaluated at our Department of Cardiovascular Medicine and had a transthoracic echocardiogram performed for suspected CS from January 1, 2005 through December 31, 2016. All CS patients were referred to echocardiographic evaluation due to symptoms (included cardiac arrhythmias, chest pain, fatigue and dyspnea), EKG alterations or for a cardiologic consult. The diagnosis of definite or probable CS was made by myocardial biopsy or in accordance with clinical criteria of the Heart Rhythm Society (HRS) consensus statement in 83 (68%) of patients; which represents our study group.<br><br>The control group consisted of 97 corrective patients with normal echocardiographic findings and without comorbidities identified during the same time frame as the CS cases.	NR
Tigen 2015	The study population consisted of 45 consecutive patients with diagnosed sarcoidosis. The diagnosis of sarcoidosis was based on ATS/ERS/WASOG statement criteria on sarcoidosis. All study population underwent a complete transthoracic echocardiography for the evaluation of cardiac functions with 2D STE.<br><br>The control group included 20 age and sex matched volunteers free of cardiovascular risk factors and without any cardiac and systemic disease.	Patients were excluded from the study if they were known to have poor echogenicity, impaired left ventricular (LV) systolic function (ejection fraction (EF) < 55 %), significant valvular disease, history of coronary artery disease, malignancy, systemic arterial hypertension, storage disorders such as Fabry’s disease, and cardiac amyloidosis. Three patients had poor echogenicity and two patients had systemic arterial hypertension.

Abbreviations: AF: atrial fibrillation; CAD: coronary artery disease; CS: cardiac sarcoidosis; DM: diabetes mellitus; ECG: electrocardiography; EF: ejection fraction; LV: left ventricular; NR: not reported; STE: speckle-tracking echocardiography; TTE: transthoracic echocardiography.

Supplementary Table S4: Quality Assessment for the included studies using the Newcastle-Ottawa Scale (NOS).

**Study ID**	**Selection**				**Comparability**	**Outcome**			**Total Score**
	Representativeness of the exposed cohort	Selection of the non-exposed cohort	Ascertainment of exposure	Outcome of interest was not present at the start of the study		Assessment of outcome	Follow-up duration	Adequacy of follow-up	
Aggeli 2013	☆	☆	☆	☆	☆☆	☆	☆	☆	9
Chen 2017	☆	☆	☆	☆	☆☆	☆	☆	☆	9
Dabir 2018		☆	☆	☆	☆☆	☆	☆	☆	8
Degirmenci 2015	☆	☆	☆	☆	☆☆	☆	☆	☆	9
Felekos 2018	☆		☆	☆	☆☆	☆	☆	☆	8
Joyce 2014	☆	☆	☆	☆	☆☆		☆	☆	8
Kul 2014	☆	☆	☆	☆	☆☆	☆	☆	☆	9
Kusunose 2020	☆	☆	☆	☆	☆☆	☆	☆	☆	9
Orii 2015	☆	☆	☆	☆	☆☆	☆	☆	☆	9
Panovsky 2021	☆	☆	☆	☆	☆☆	☆	☆	☆	9
Schouver 2020	☆	☆	☆	☆	☆	☆	☆	☆	8
Stefano 2020	☆	☆	☆	☆	☆☆	☆	☆	☆	9
Tigen 2015	☆	☆	☆	☆	☆	☆		☆	7

Supplementary Figure S1. Sensitivity analysis for LV GLS.

Study or Subgroup	Sarcoid Mean	Sarcoid SD	Sarcoid Total	Control Mean	Control SD	Control Total	Weight	Mean Difference IV, Random, 95% CI
Aggeli 2013	14.41	3.01	67	22.93	2.28	29	0.0%	−8.52 [−9.62, −7.42]
Chen 2017	16.8	5.05	54	20.1	3.2	54	7.8%	−3.30 [−4.89, −1.71]
Dabir 2018	18.6	3.9	38	21.1	3.4	22	7.2%	−2.50 [−4.39, −0.61]
Degirmenci 2015	19.1	1.4	50	20.8	1.4	50	9.6%	−1.70 [−2.25, −1.15]
Felekos 2018	15.1	3.1	107	20.9	2.3	45	9.2%	−5.80 [−6.69, −4.91]
Joyce 2014	17.3	2.5	100	20	1.6	100	9.6%	−2.70 [−3.28, −2.12]
Kul 2014	17.6	1.9	34	20.8	1.9	26	9.0%	−3.20 [−4.17, −2.23]
Kusunose 2020	16.6	2.4	101	19.5	2.7	52	9.2%	−2.90 [−3.77, −2.03]
Orii 2015	22	5	32	23	5	10	4.2%	−1.00 [−4.55, 2.55]
Panovsky 2021	13.9	3.1	110	14.2	2.5	22	8.6%	−0.30 [−1.49, 0.89]
Schouver 2017	17.2	3.1	35	21.3	1.5	35	8.7%	−4.10 [−5.24, −2.96]
Stefano 2020	12.4	3.7	83	18.2	2.7	97	9.1%	−5.80 [−6.76, −4.84]
Tigen 2015	21.9	3.3	40	25.7	3	20	7.7%	−3.80 [−5.47, −2.13]
**Total (95% CI)**			**784**			**533**	**100.0%**	**−3.19 [−4.15, −2.23]**

Heterogeneity: $Tau^2 = 2.42$; $Chi^2 = 118.89$, df = 11 ($P < 0.00001$); $I^2 = 91\%$
Test for overall effect: Z = 6.50 ($P < 0.00001$)

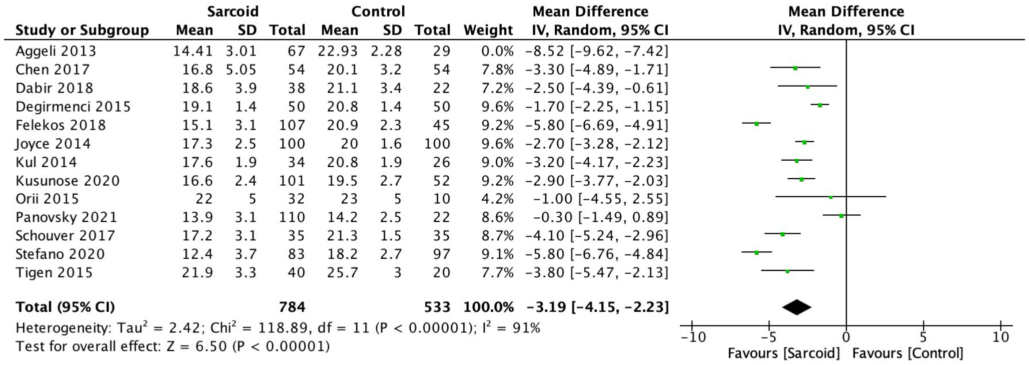

Supplementary Figure S2. Sensitivity analysis for LV GCS.

Study or Subgroup	Sarcoid Mean	Sarcoid SD	Sarcoid Total	Control Mean	Control SD	Control Total	Weight	Mean Difference IV, Random, 95% CI
Kul 2014	19	2.7	34	22.7	3.1	26	24.4%	−3.70 [−5.20, −2.20]
Orii 2015	28	7	32	30	7	10	6.4%	−2.00 [−6.97, 2.97]
Panovsky 2021	23.4	4	110	22.2	2.9	22	0.0%	1.20 [−0.22, 2.62]
Schouver 2017	19.9	4.3	35	21.3	1.5	35	24.3%	−1.40 [−2.91, 0.11]
Stefano 2020	17.1	6.5	83	21.9	4.7	97	22.8%	−4.80 [−6.48, −3.12]
Tigen 2015	21.1	3.6	40	25.4	3.1	20	22.1%	−4.30 [−6.06, −2.54]
**Total (95% CI)**			**224**			**188**	**100.0%**	**−3.41 [−4.81, −2.02]**

Heterogeneity: $Tau^2 = 1.47$; $Chi^2 = 10.88$, df = 4 ($P = 0.03$); $I^2 = 63\%$
Test for overall effect: Z = 4.81 ($P < 0.00001$)

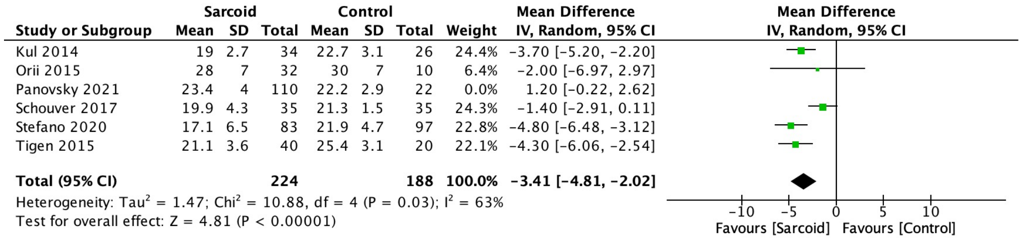

Supplementary Figure S3. Sensitivity analysis for LVEF.

Study or Subgroup	Sarcoid Mean	Sarcoid SD	Sarcoid Total	Control Mean	Control SD	Control Total	Weight	Mean Difference IV, Random, 95% CI
Aggeli 2013	58.73	4.49	67	59.34	3.95	29	9.3%	−0.61 [−2.41, 1.19]
Chen 2017	58.4	8.5	54	59.9	9.7	54	7.0%	−1.50 [−4.94, 1.94]
Dabir 2018	64.9	5	38	63.8	9	22	6.2%	1.10 [−2.98, 5.18]
Degirmenci 2015	64.1	2.6	50	67.8	2.4	50	10.2%	−3.70 [−4.68, −2.72]
Felekos 2018	59.1	2.4	107	58.7	4.5	45	9.8%	0.40 [−0.99, 1.79]
Joyce 2014	57	5	100	63	6	100	9.6%	−6.00 [−7.53, −4.47]
Kul 2014	64.6	9.4	34	63.4	7.4	26	6.0%	1.20 [−3.05, 5.45]
Kusunose 2020	65	5	101	64	4	52	9.7%	1.00 [−0.46, 2.46]
Orii 2015	61	4	32	61	2.7	10	8.8%	0.00 [−2.17, 2.17]
Panovsky 2021	66	7	113	65	5	22	8.4%	1.00 [−1.46, 3.46]
Schouver 2017	59	12.2	35	57.7	3.6	35	6.0%	1.30 [−2.91, 5.51]
Stefano 2020	43.2	12.4	83	63.9	3.8	97	0.0%	−20.70 [−23.47, −17.93]
Tigen 2015	71.1	5.3	40	73.6	3.2	20	8.8%	−2.50 [−4.66, −0.34]
**Total (95% CI)**			**771**			**465**	**100.0%**	**−0.90 [−2.48, 0.68]**

Heterogeneity: $Tau^2 = 6.09$; $Chi^2 = 82.65$, df = 11 ($P < 0.00001$); $I^2 = 87\%$
Test for overall effect: Z = 1.12 ($P = 0.26$)

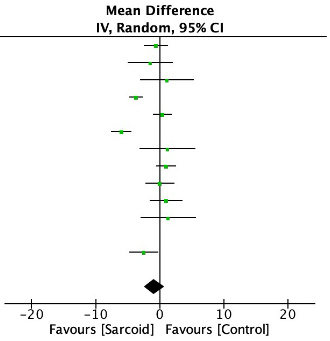

Supplementary Figure S4. Sensitivity analysis for LV GRS.

	Sarcoid			Control				Mean Difference
Study or Subgroup	Mean	SD	Total	Mean	SD	Total	Weight	IV, Random, 95% CI
Orii 2015	45	23	32	46	18	10	11.4%	−1.00 [−14.71, 12.71]
Panovsky 2021	53.4	13.5	110	51.2	13.6	22	36.5%	2.20 [−4.02, 8.42]
Stefano 2020	29.3	12.8	83	44.1	12.1	97	0.0%	−14.80 [−18.46, −11.14]
Tigen 2015	45	11	40	49.7	5.9	20	52.1%	−4.70 [−8.98, −0.42]
**Total (95% CI)**			**182**			**52**	**100.0%**	**−1.76 [−6.74, 3.21]**

Heterogeneity: $Tau^2 = 7.60$; $Chi^2 = 3.25$, df = 2 (P = 0.20); $I^2 = 39\%$
Test for overall effect: Z = 0.69 (P = 0.49)

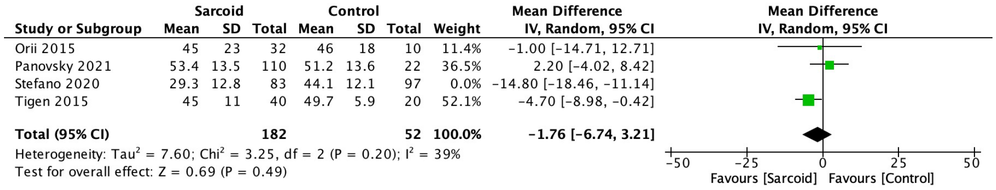

Supplementary Figure S5. Sensitivity analysis for IVST.

	Sarcoid			Control				Mean Difference
Study or Subgroup	Mean	SD	Total	Mean	SD	Total	Weight	IV, Random, 95% CI
Aggeli 2013	8.7	1.03	67	9.1	1.07	29	16.2%	−0.40 [−0.86, 0.06]
Chen 2017	11.1	2.7	54	10.3	1.6	54	11.8%	0.80 [−0.04, 1.64]
Dabir 2018	9.5	1.7	38	9.7	1.9	22	10.5%	−0.20 [−1.16, 0.76]
Degirmenci 2015	9	1	50	8	0.9	50	0.0%	1.00 [0.63, 1.37]
Felekos 2018	8.8	1.1	107	9.2	1.05	45	17.1%	−0.40 [−0.77, −0.03]
Joyce 2014	9.6	1.8	100	9.2	1.9	100	15.6%	0.40 [−0.11, 0.91]
Kul 2014	9	1	34	8	1	26	15.6%	1.00 [0.49, 1.51]
Orii 2015	8	1	32	8	1	10	13.2%	0.00 [−0.71, 0.71]
**Total (95% CI)**			**432**			**286**	**100.0%**	**0.16 [−0.30, 0.62]**

Heterogeneity: $Tau^2 = 0.29$; $Chi^2 = 27.75$, df = 6 (P = 0.0001); $I^2 = 78\%$
Test for overall effect: Z = 0.67 (P = 0.50)

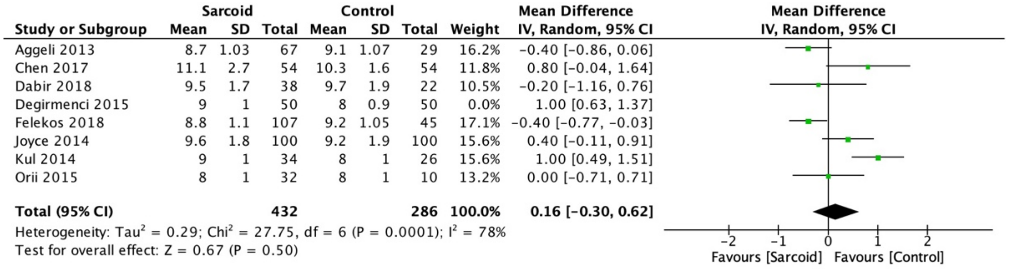

Supplementary Figure S6. Sensitivity analysis for TAPSE (omitting Joyce 2014).

	Sarcoid			Control				Mean Difference
Study or Subgroup	Mean	SD	Total	Mean	SD	Total	Weight	IV, Random, 95% CI
Joyce 2014	21	3.5	100	23	3.7	100	0.0%	−2.00 [−3.00, −1.00]
Kul 2014	23	5.1	34	24	3.6	26	31.1%	−1.00 [−3.20, 1.20]
Kusunose 2020	21	3	101	21	5	52	68.9%	0.00 [−1.48, 1.48]
**Total (95% CI)**			**135**			**78**	**100.0%**	**−0.31 [−1.54, 0.92]**

Heterogeneity: $Tau^2 = 0.00$; $Chi^2 = 0.55$, df = 1 (P = 0.46); $I^2 = 0\%$
Test for overall effect: Z = 0.50 (P = 0.62)

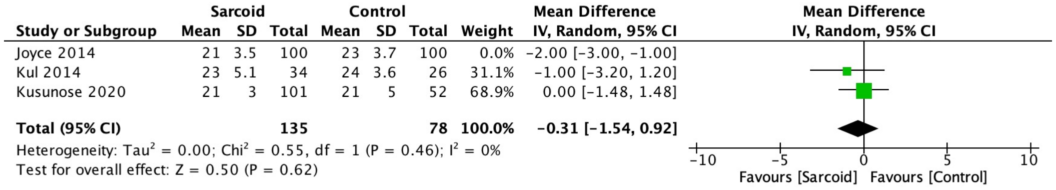

Supplementary Figure S7. Sensitivity analysis for TAPSE (omitting Kusunose 2020).

Study or Subgroup	Sarcoid Mean	Sarcoid SD	Sarcoid Total	Control Mean	Control SD	Control Total	Weight	Mean Difference IV, Random, 95% CI
Joyce 2014	21	3.5	100	23	3.7	100	83.0%	−2.00 [−3.00, −1.00]
Kul 2014	23	5.1	34	24	3.6	26	17.0%	−1.00 [−3.20, 1.20]
Kusunose 2020	21	3	101	21	5	52	0.0%	0.00 [−1.48, 1.48]
**Total (95% CI)**			**134**			**126**	**100.0%**	**−1.83 [−2.74, −0.92]**

Heterogeneity: $Tau^2 = 0.00$; $Chi^2 = 0.66$, df = 1 (P = 0.42); $I^2 = 0\%$
Test for overall effect: Z = 3.94 (P < 0.0001)

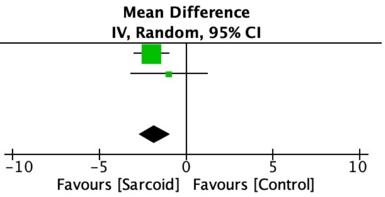

Supplementary Figure S8. Sensitivity analysis for PASP.

Study or Subgroup	Sarcoid Mean	Sarcoid SD	Sarcoid Total	Control Mean	Control SD	Control Total	Weight	Mean Difference IV, Random, 95% CI
Felekos 2018	27.9	3.9	107	26.4	3.3	45	0.0%	1.50 [0.29, 2.71]
Joyce 2014	25	15	100	18	10	100	73.5%	7.00 [3.47, 10.53]
Schouver 2017	27.5	15.9	35	22.4	7.9	35	26.5%	5.10 [−0.78, 10.98]
**Total (95% CI)**			**135**			**135**	**100.0%**	**6.50 [3.47, 9.53]**

Heterogeneity: $Tau^2 = 0.00$; $Chi^2 = 0.29$, df = 1 (P = 0.59); $I^2 = 0\%$
Test for overall effect: Z = 4.20 (P < 0.0001)

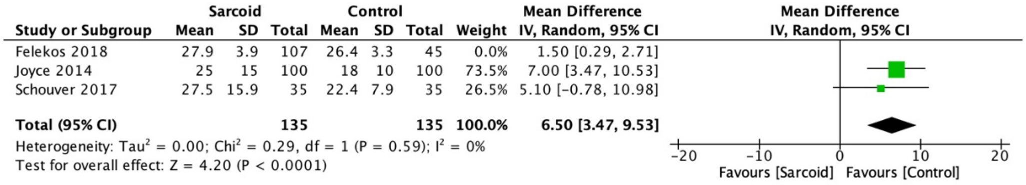

Supplementary Figure S9. Sensitivity analysis for LVESD.

Study or Subgroup	Sarcoid Mean	Sarcoid SD	Sarcoid Total	Control Mean	Control SD	Control Total	Weight	Mean Difference IV, Random, 95% CI
Aggeli 2013	24.2	3	67	23.1	3.2	29	23.9%	1.10 [−0.27, 2.47]
Felekos 2018	24.6	2.7	107	24.3	3.1	45	41.4%	0.30 [−0.74, 1.34]
Joyce 2014	32	5	100	32	4	100	28.4%	0.00 [−1.25, 1.25]
Kul 2014	30	4	34	28	6	26	6.3%	2.00 [−0.67, 4.67]
Tigen 2015	28	3	40	26	2	20	0.0%	2.00 [0.72, 3.28]
**Total (95% CI)**			**308**			**200**	**100.0%**	**0.51 [−0.16, 1.18]**

Heterogeneity: $Tau^2 = 0.00$; $Chi^2 = 2.70$, df = 3 (P = 0.44); $I^2 = 0\%$
Test for overall effect: Z = 1.50 (P = 0.13)

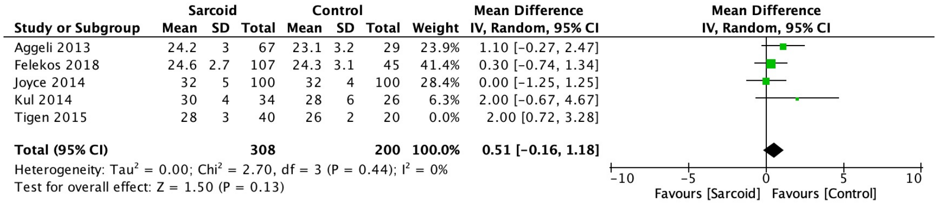

Supplementary Figure S10. Sensitivity analysis for E-wave velocity.

	Sarcoid			Control				Mean Difference
Study or Subgroup	Mean	SD	Total	Mean	SD	Total	Weight	IV, Random, 95% CI
Aggeli 2013	80	30	67	70	20	29	14.0%	10.00 [−0.23, 20.23]
Chen 2017	76	28.3	54	83.9	20.9	54	15.0%	−7.90 [−17.28, 1.48]
Degirmenci 2015	78.6	16	50	90	12.8	50	19.4%	−11.40 [−17.08, −5.72]
Felekos 2018	75.2	5.9	107	70	4.1	45	0.0%	5.20 [3.56, 6.84]
Joyce 2014	76	17	100	76	19	100	20.2%	0.00 [−5.00, 5.00]
Kul 2014	77	17	34	78	21	26	14.4%	−1.00 [−10.89, 8.89]
Tigen 2015	80	20	40	90	10	20	17.1%	−10.00 [−17.59, −2.41]
**Total (95% CI)**			**345**			**279**	**100.0%**	**−3.84 [−9.89, 2.20]**

Heterogeneity: $Tau^2 = 40.67$; $Chi^2 = 19.51$, df = 5 (P = 0.002); $I^2 = 74\%$
Test for overall effect: Z = 1.25 (P = 0.21)

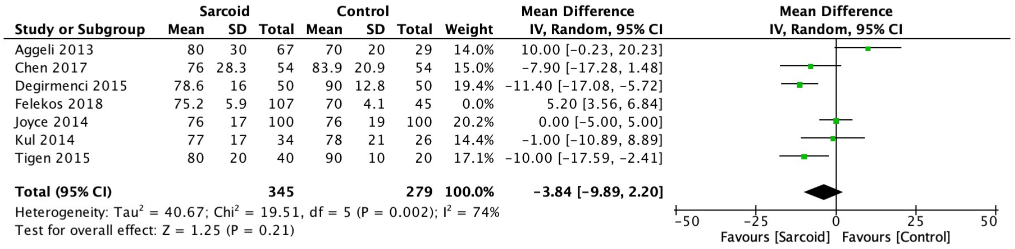

Supplementary Figure S11. Sensitivity analysis for A-wave velocity.

	Sarcoid			Control				Mean Difference
Study or Subgroup	Mean	SD	Total	Mean	SD	Total	Weight	IV, Random, 95% CI
Aggeli 2013	60	20	67	55	10	29	14.7%	5.00 [−1.02, 11.02]
Chen 2017	70.4	28.3	54	71.4	21.1	54	6.0%	−1.00 [−10.42, 8.42]
Degirmenci 2015	72.2	20.1	50	62.3	9	50	0.0%	9.90 [3.80, 16.00]
Felekos 2018	57.3	9.4	107	55.6	10.3	45	43.4%	1.70 [−1.80, 5.20]
Joyce 2014	70	16	100	70	18	100	23.8%	0.00 [−4.72, 4.72]
Kul 2014	67	16	34	66	17	26	7.4%	1.00 [−7.46, 9.46]
Tigen 2015	80	20	40	70	20	20	4.6%	10.00 [−0.74, 20.74]
**Total (95% CI)**			**402**			**274**	**100.0%**	**1.95 [−0.36, 4.25]**

Heterogeneity: $Tau^2 = 0.00$; $Chi^2 = 4.25$, df = 5 (P = 0.51); $I^2 = 0\%$
Test for overall effect: Z = 1.66 (P = 0.10)

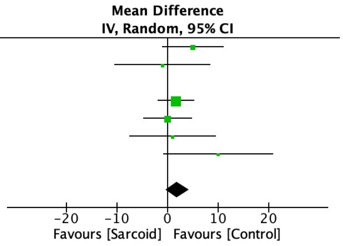

Supplementary Figure S12. Sensitivity analysis for E/A ratio.

	Sarcoid			Control				Mean Difference
Study or Subgroup	Mean	SD	Total	Mean	SD	Total	Weight	IV, Random, 95% CI
Aggeli 2013	1.093	0.29	67	1.1	0.4	29	12.0%	−0.01 [−0.17, 0.15]
Chen 2017	1.17	0.47	54	1.23	0.46	54	10.3%	−0.06 [−0.24, 0.12]
Degirmenci 2015	1.07	0.32	50	1.2	0.36	50	16.7%	−0.13 [−0.26, 0.00]
Joyce 2014	1.1	0.3	100	1.06	0.32	100	33.6%	0.04 [−0.05, 0.13]
Kul 2014	1.38	1.11	34	1.2	0.39	26	2.1%	0.18 [−0.22, 0.58]
Kusunose 2020	0.94	0.36	101	0.89	0.33	52	21.8%	0.05 [−0.06, 0.16]
Schouver 2017	1.3	0.8	35	1.2	0.5	35	3.4%	0.10 [−0.21, 0.41]
Stefano 2020	1.19	0.63	83	1.6	0.6	97	0.0%	−0.41 [−0.59, −0.23]
**Total (95% CI)**			**441**			**346**	**100.0%**	**0.00 [−0.06, 0.06]**

Heterogeneity: $Tau^2 = 0.00$; $Chi^2 = 6.80$, df = 6 (P = 0.34); $I^2 = 12\%$
Test for overall effect: Z = 0.09 (P = 0.92)

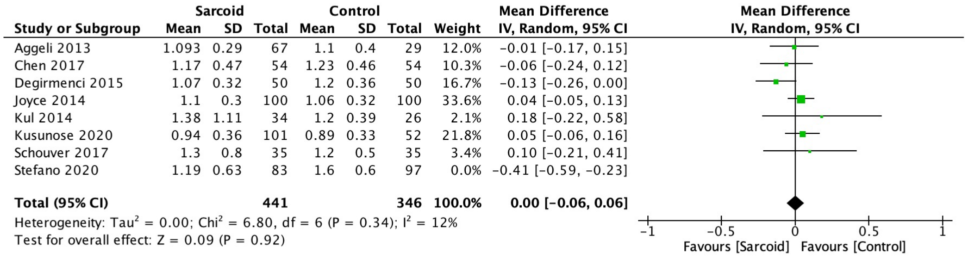

Supplementary Figure S13. Sensitivity analysis for E/E’ ratio.

	Sarcoid			Control				Mean Difference
Study or Subgroup	Mean	SD	Total	Mean	SD	Total	Weight	IV, Random, 95% CI
Aggeli 2013	7	1.45	67	6.4	1.39	29	28.9%	0.60 [−0.01, 1.21]
Chen 2017	9.09	5.11	54	9.72	3.79	54	8.7%	−0.63 [−2.33, 1.07]
Joyce 2014	12	4	100	11	4	100	16.1%	1.00 [−0.11, 2.11]
Kul 2014	8.5	2.5	34	7.7	2.5	26	13.4%	0.80 [−0.48, 2.08]
Kusunose 2020	10.1	5.3	101	8.7	2.8	52	13.3%	1.40 [0.12, 2.68]
Schouver 2017	9	1.8	35	7.2	2.2	35	19.6%	1.80 [0.86, 2.74]
Stefano 2020	13.4	10.1	83	7.7	1.9	97	0.0%	5.70 [3.49, 7.91]
**Total (95% CI)**			**391**			**296**	**100.0%**	**0.93 [0.37, 1.48]**

Heterogeneity: $Tau^2 = 0.18$; $Chi^2 = 8.19$, df = 5 (P = 0.15); $I^2 = 39\%$
Test for overall effect: Z = 3.25 (P = 0.001)

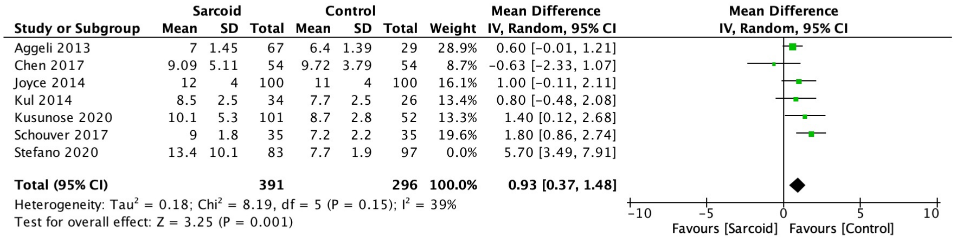

Supplementary Figure S14. Funnel plot for LV GLS.

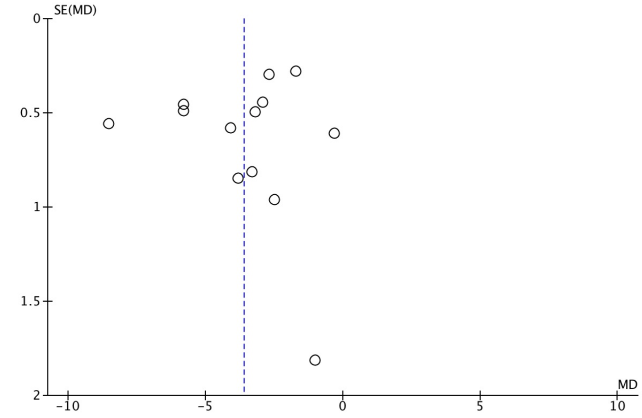

Supplementary Figure S15. Funnel plot for LV GCS.

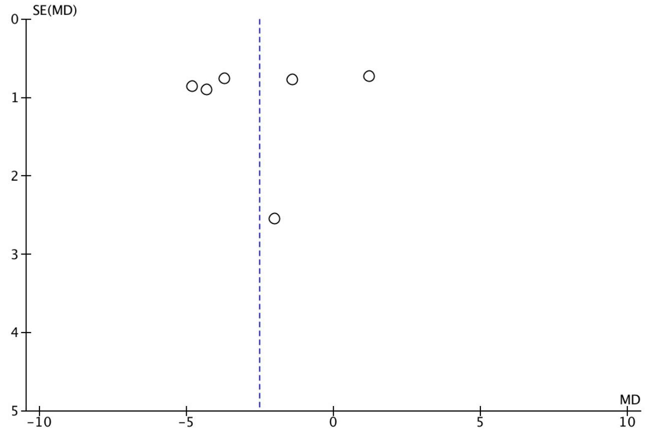

Supplementary Figure S16. Funnel plot for LVEF.

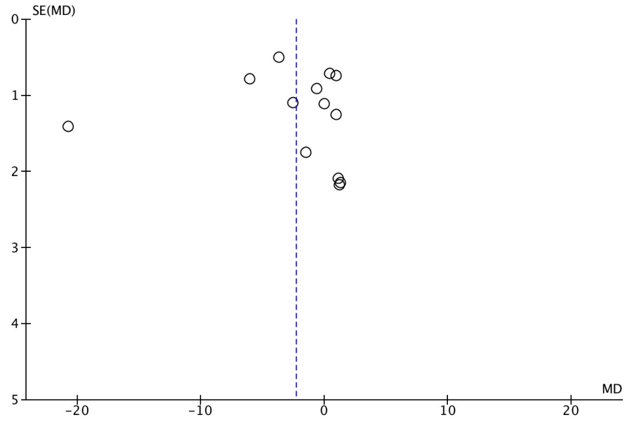

Supplementary Figure S17. Funnel plot for LV GRS.

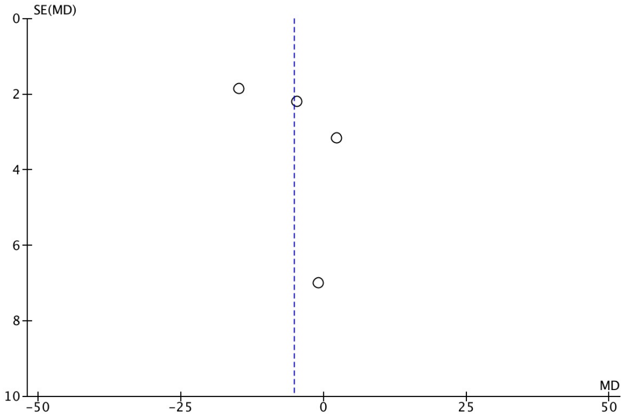

Supplementary Figure S18. Funnel plot for IVST.

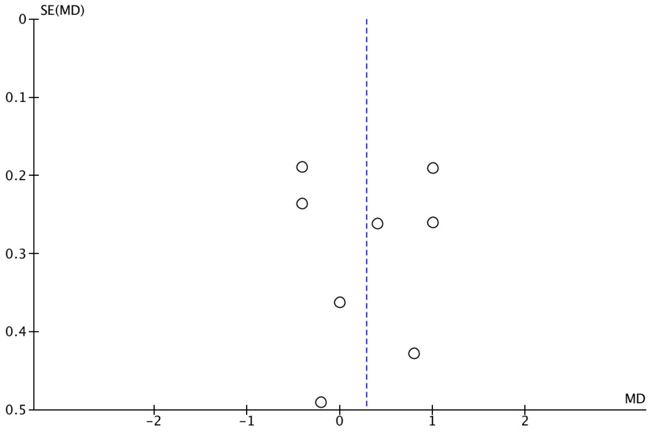

Supplementary Figure S19. Funnel plot for TAPSE.

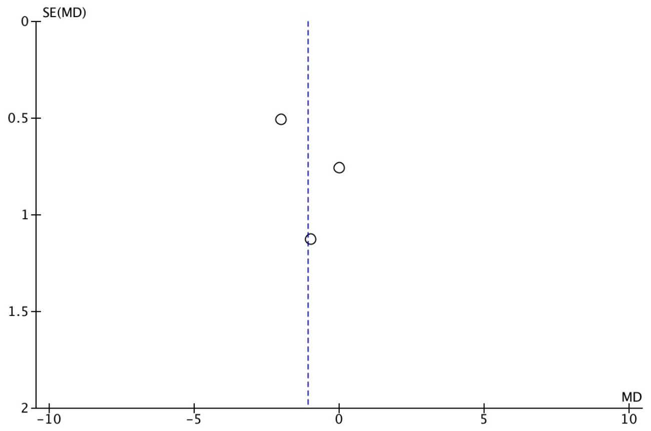

Supplementary Figure S20. Funnel plot for PASP.

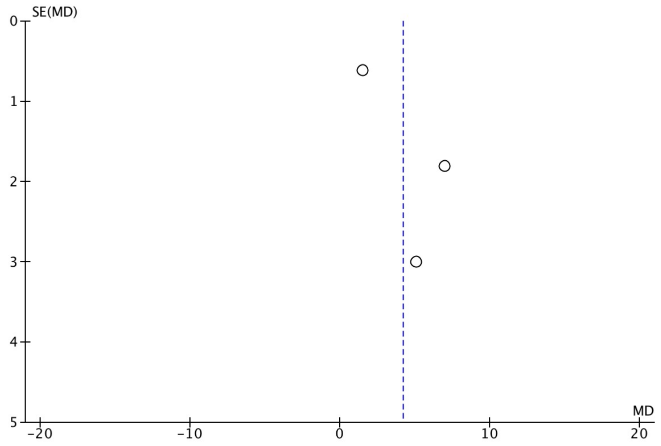

Supplementary Figure S21. Funnel plot for LVEDD.

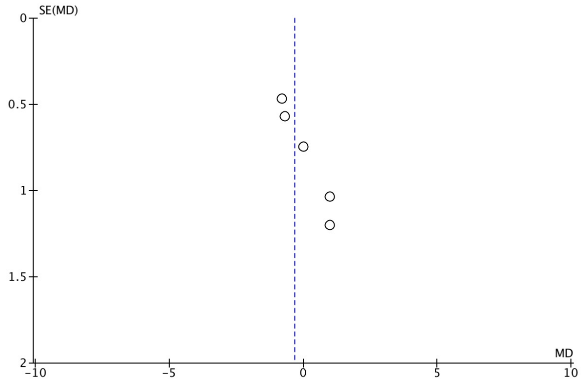

Supplementary Figure S22. Funnel plot for LVESD.

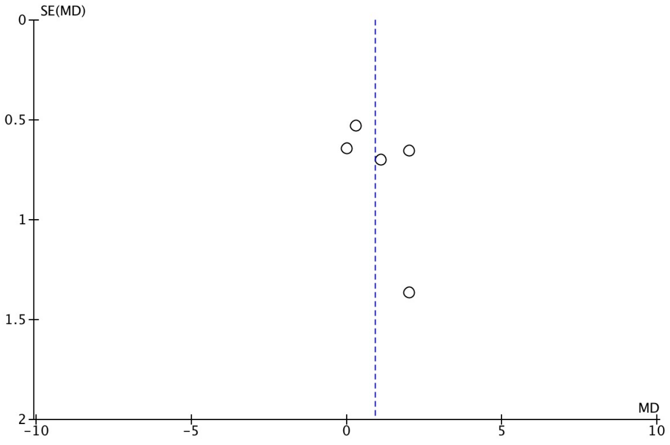

Supplementary Figure S23. Funnel plot for E-wave velocity.

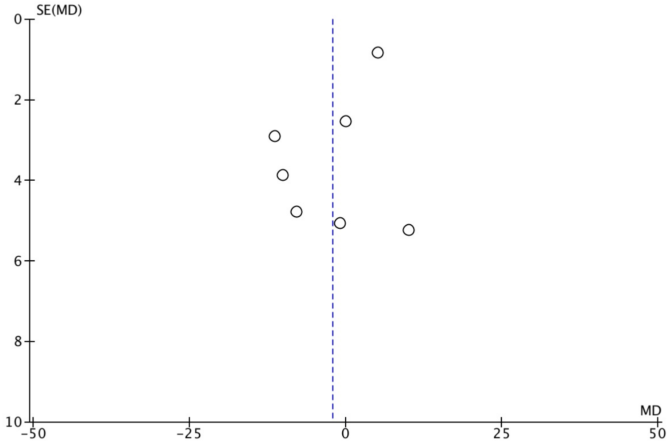

Supplementary Figure S24. Funnel plot for A-wave velocity.

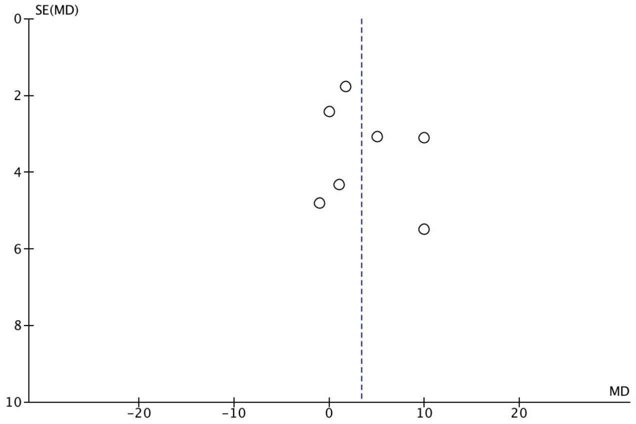

Supplementary Figure S25. Funnel plot for E/A ratio.

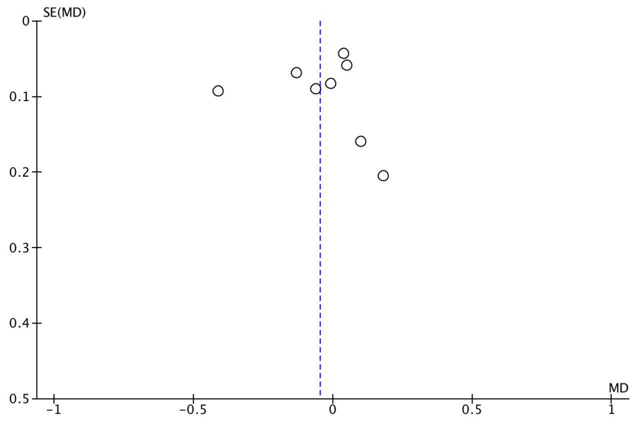

Supplementary Figure S26. Funnel plot for E/E' ratio.

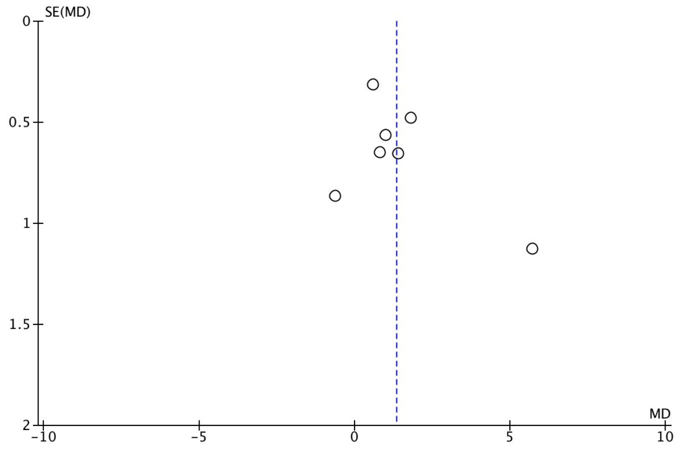

Supplement: Supplementary file 1 [file diagnostics-15-00708-s001.zip › diagnostics-3464119-supplementary.pdf]
